# Supplementary material for: Blockade of IGF2R improves muscle regeneration and ameliorates Duchenne muscular dystrophy
Source: EMBO Mol Med. 2019 Dec 2;12(1):e11019. doi: 10.15252/emmm.201911019 (PMC6949491; doi:10.15252/emmm.201911019)
Supplement: Supplementary file 1 — Appendix [file EMMM-12-e11019-s001.pdf]

APPENDIX

Table of content

Table of content..1

Appendix Figure S1...2

Appendix Figure S2...3

Appendix Table S1.. 4

Appendix Table S2.. 5

Appendix Figure S1. (a) Size distribution of anti IGFR2 aggregates was detected by DLS technique. N=3 different measurements (red, blue, and green lines) were acquired. The graph shows two major peaks in the ranges of 10-20 nm (centered at 12.8nm ±3.77) and 180-200nm (centered at 196.8nm ±56), with an intensity percentage of 71.5% and the 28.5%, respectively. PBS1X did not shows notable peaks. (b) NTA detection of anti IGF2R aggregate size distribution. N=5 measurements were acquired and averaged (red lines). Since PBS 1X, used as diluent, presents barely detectable peaks below100nm, the graph region showing peaks between 0 and 80 nm is likely owned only to IGFR2 formulation. Histogram of anti IGF2R particle concentration in the main regions between 0 and 80 nm (One-way ANOVA, p>0.1). (c) Analysis of reactivity against polyclonal goat anti IGF2R antibody in plasma samples collected from high and low dose injected mice treated for 4 and 9 weeks. Reactivity was expressed as fold change over the untreated (n.i.) mouse values. Not significant differences were measured between the samples (One-way ANOVA, p>0.05). (d) Detection of light (23 kDa) and heavy (53.6 kDa) chain goat IgG presence in TAs and VMs from high and low dose anti-IGF2R injected mice. Untreated mdx and C57Bl murine muscles were used as controls. Ponceau staining was used for WB lane quantification.

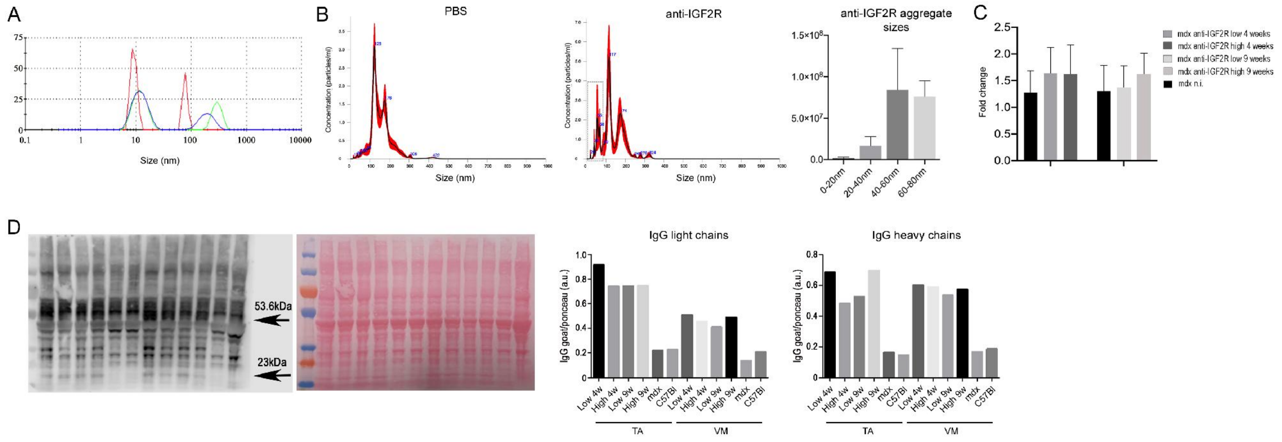

Appendix Figure S2. Diagram representing the lentiviral vectors with the RFP-Puromycin reporter gene expressed under a Rous sarcoma virus [Rsv] promoter

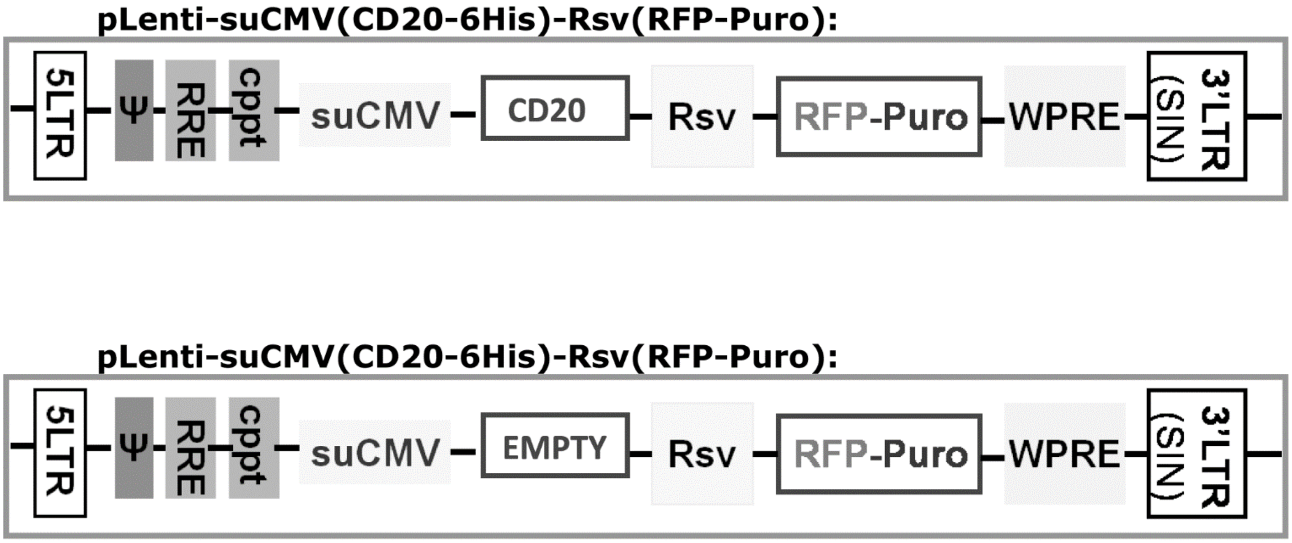

Appendix Table S1. Primer sequence for RT-qPCR

**Gene**	**Forward (5'-3')**	**Reverse (5'-3')**
β-actin	TGGCACCACACCTTCTACAATGAG	CCGTGGTGGTGAAGCTGTAGCC
IGF2	TCTCGAGGCACCCTAAATTACC	TCAGCAAATGCCCCTGAAAG
IGF1	AGAAGTGTGAGTCTCTGTTCC	GCCAGCCTCTAAAAGATTCTG
IGF1Rβ	ACACACACACACACACAC	CAGACAACTCCAGCTTTCC

Appendix Table S1. Exact p-values

Figure		Exact p value	Statistical test
1	A	Ratio IGF2/β-actin:<br>C2C12+IGF1/C2C12:****<0.0001<br>C2C12 OECD20+IGF1/C2C12:****<0.0001<br>C2C12 OECD20+IGF1/C2C12+IGF1:****<0.0001	One-way ANOVA
	C	CD20 immunoprecipitation using anti-pSer+pThr in C2C12 cells treated with IGF1 1nM ON/treated with IGF1 1nM 2h: *= 0.03205<br>CD20 immunoprecipitation using anti-pSer+pThr in C2C12 cells treated with IGF1 1nM ON/untreated C2C12: *= 0.03741<br>CD20 immunoprecipitation using anti-pSer+pThr in C2C12 cells treated with IGF1 10nM nM 2h/untreated C2C12: **= 0.0046	Two-way ANOVA
	D	CD20 immunoprecipitation using anti-pSer+pThr in ShCTR-treated C2C12 cells treated with IGF1 10nM and anti-IGF2R/treated with IGF1 10 nM: ****<0.0001<br>CD20 immunoprecipitation using anti-pSer+pThr in ShCTR-treated C2C12 cells treated with IGF1 10nM and anti-IGF2R/treated with anti-FLAG: ****<0.0001<br>CD20 immunoprecipitation using anti-pSer+pThr in ShCTR-treated C2C12 cells treated with IGF1 10nM and anti-IGF2R/ShCD20-treated C2C12 cells treated with IGF1 10nM and anti-IGF2R: *= 0.0412<br><br>CD20 immunoprecipitation using anti-pSer+pThr in ShCD20-treated C2C12 cells treated with IGF1 10nM and anti-IGF2R/treated with IGF1 10 nM: ****<0.0001<br>CD20 immunoprecipitation using anti-pSer+pThr in ShCD20-treated C2C12 cells treated with IGF1 10nM and anti-IGF2R/treated with anti-FLAG: ****<0.0001	Two-way ANOVA
	E	CD20 immunoprecipitation using anti-pSer+pThr in C2C12 treated with IGF1 1nM/untreated C2C12: ****<0.0001<br>CD20 immunoprecipitation using anti-pSer+pThr in C2C12 treated with IGF1 10nM and IGF2 100ng/ml/untreated C2C12: ****<0.0001<br>CD20 immunoprecipitation using anti-pSer+pThr in C2C12 treated with IGF1 1nM and IGF2 10ng/ml/untreated C2C12: ****<0.0001	Two-way ANOVA
	F	IGF2R/ β-actin<br>Untreated C2C12/C2C12 + anti-IGF2R: *= 0.0293	Student t-Test
2	G	Fusion index:<br>Untreated C2C12/ShCD20+anti-IGF2R C2C12: ***= 0.00032<br>C2C12 + anti-IGF2R/untreated C2C12: **= 0.0064<br>C2C12 + anti-IGF2R/ShCD20+anti-IGF2R C2C12: ****<0.0001<br>HsKM+anti-IGF2R/untreated C2C12: *= 0.0421<br>HsKM+anti-IGF2R/ ShCD20+anti-IGF2R C2C12: ***= 0.00098	Two-way ANOVA
2	H	Myog/ β-actin<br>C2C12 shCD20+anti-IGF2R 2 days DM/C2C12 untreated 2 days DM: ****<0.0001<br>C2C12 shCD20+anti-IGF2R 4 days DM/C2C12 untreated 4 days DM: **= 0.0100<br>C2C12 +anti-IGF2R 2 days DM/C2C12 untreated 2 days DM: **= 0.00841<br>C2C12 +anti-IGF2R 4 days DM/C2C12 untreated 4 days DM: ****<0.0001<br>C2C12 +anti-IGF2R 6 days DM/ShCD20-treated C2C12 untreated 6 days DM: *= 0.0193<br>P.M. HsKM+anti-IGF2R/P.M. C2C12 untreated: **= 0.00651<br><br>Myf5/ β-actin<br>C2C12 shCD20+anti-IGF2R 4 days DM/C2C12 untreated 4 days DM: *= 0.0411<br>P.M. C2C12 +anti-IGF2R/C2C12 untreated: *= 0.0483<br>P.M. C2C12 +anti-IGF2R/ShCD20-treated C2C12 untreated: **= 0.0099<br>C2C12 +anti-IGF2R 2 days DM/ ShCD20-treated C2C12 untreated 2 days DM: **= 0.0074<br>C2C12 +anti-IGF2R 4 days DM/C2C12 untreated 4 days DM: ***= 0.00077<br>C2C12 +anti-IGF2R 4 days DM/ShCD20-treated C2C12 untreated 4 days DM: ***= 0.00069<br><br>MyHC/ β-actin	Two-way ANOVA

		C2C12 +anti-IGF2R 2 days DM/C2C12 untreated 2 days DM: ***= 0.0004<br>C2C12 +anti-IGF2R 2 days DM/ShCD20-treated C2C12 untreated 2 days DM: ***= 0.00088<br>C2C12 +anti-IGF2R 4 days DM/C2C12 untreated 4 days DM: ****<0.0001<br>C2C12 +anti-IGF2R 4 days DM/ShCD20-treated C2C12 untreated 4 days DM: ****<0.0001 C2C12 +anti-IGF2R 6 days DM/C2C12 untreated 6 days DM: *= 0.0311	
3	D	pSer+pThr/CD20<br>TA mdx/TA C57BL6/J: ****<0.0001<br>VM mdx/VM C57BL6/J: ****<0.0001	One-way ANOVA
	E	IGF2R/GAPDH<br>TA mdx/TA C57BL6/J: ****<0.0001<br>TA DMD patients/TA healthy volunteers: ****<0.0001<br>VM mdx/VM C57BL6/J: ****<0.0001<br>VM DMD patients/VM healthy volunteers: ***=0.0001	One-way ANOVA
	F	IGF2R fold increase<br>TA mdx/TA C57BL6/J: *=0.0392<br>TA DMD patients/TA healthy volunteers: ****<0.0001<br>VM mdx/VM C57BL6/J: ****<0.0001<br>VM DMD patients/VM healthy volunteers: ***=0.0001	One-way ANOVA
4	B	Regenerating fibers/total fibers<br>VM mdx anti-IGF2R High 9w/untreated mdx: ****<0.0001<br>VM mdx anti-IGF2R Low 9w/untreated mdx: ****<0.0001<br><br>mCad+ cells/section<br>TA mdx+anti-IGF2R High 9w/untreated mdx: ****<0.0001<br>TA mdx+anti-IGF2R Low 9w/untreated mdx: ****<0.0001<br>TA mdx+anti-IGF2R High 4w/untreated mdx: ***= 0.00033<br>TA mdx+anti-IGF2R Low 4w/untreated mdx: **= 0.0096<br>VM mdx+anti-IGF2R High 9w/untreated mdx: ****<0.0001<br>VM mdx+anti-IGF2R Low 9w/untreated mdx: ****<0.0001<br><br>N=10	One-way ANOVA
4	C	Neonatal MHC/ β-actin<br>TA mdx High 4w/untreated mdx: ****<0.0001<br>TA mdx Low 4w/untreated mdx: ****<0.0001<br>TA mdx High 9w/untreated mdx: ****<0.0001<br>TA mdx Low 9w/untreated mdx: ****<0.0001<br><br>VM mdx High 4w/untreated mdx: ****<0.0001<br>VM mdx Low 4w/untreated mdx: ****<0.0001<br>VM mdx High 9w/untreated mdx: ****<0.0001<br>VM mdx Low 9w/untreated mdx: **=0.0073<br><br>N=10	One-way ANOVA
4	D	Po/CSA 4 weeks of anti-IGF2R treatment<br>TA C57BL6/J/mdx untreated: **= 0.0065<br>TA mdx Low dose/mdx untreated: ***= 0.00028<br>TA mdx High dose/mdx untreated: **= 0.0088<br>VM C57BL6/J/mdx untreated: *= 0.0433<br>VM mdx Low dose/mdx untreated: ***= 0.00052<br><br>Po/CSA 9 weeks of anti-IGF2R treatment<br>TA C57BL6/J/mdx untreated: **= 0.0084<br>VM C57BL6/J/mdx untreated: *= 0.0368<br>VM mdx High dose/mdx untreated: **= 0.0048<br><br>N=10	One-way ANOVA
5	A	48 kDa calcineurin A<br>TA mdx +anti-IGF2R High 4w/untreated mdx: *=0.0284<br>60 kDa calcineurin A	Two-way ANOVA

		TA mdx untreated/mdx +anti-IGF2R High 4w: *=0.0311<br><br>48 kDa calcineurin A<br>VM mdx +anti-IGF2R High 4w/untreated mdx: ****<0.0001<br>VM mdx +anti-IGF2R High 9w/untreated mdx: ****<0.0001<br>VM mdx +anti-IGF2R Low 4w/untreated mdx: ****<0.0001<br>VM mdx +anti-IGF2R Low 9w/untreated mdx: ****<0.0001<br>VM mdx untreated/C57BL6/J: ****<0.0001<br>60 kDa calcineurin A<br>VM mdx untreated/mdx +anti-IGF2R High 4w: ****<0.0001<br>VM mdx untreated/mdx +anti-IGF2R High 9w: ****<0.0001<br>VM mdx untreated/mdx +anti-IGF2R Low 4w: ****<0.0001<br>VM mdx untreated/mdx +anti-IGF2R Low 9w: **= 0.0065<br>VM C57BL6/J/mdx untreated: ****<0.0001<br><br>N=10	
	B	Ratio pCAMKII/CAMKII<br>TA C57Bl6/J/mdx +anti-IGF2R High 4w: *= 0.04534<br>TA C57Bl6/J/mdx +anti-IGF2R High 9w: *= 0.03898<br>TA C57Bl6/J/mdx +anti-IGF2R Low 4w: *= 0.04883<br>TA C57Bl6/J/mdx +anti-IGF2R Low 9w: *= 0.01084<br>TA mdx untreated/mdx +anti-IGF2R High 4w: **= 0.01<br>TA mdx untreated/mdx +anti-IGF2R High 9w: *= 0.0209<br>TA mdx untreated/mdx +anti-IGF2R Low 4w: **= 0.0095<br>TA mdx untreated/mdx +anti-IGF2R Low 9w: **= 0.0085<br><br>VM C57Bl6/J/mdx +anti-IGF2R High 4w: *= 0.0453<br>VM C57Bl6/J/mdx +anti-IGF2R High 9w: *= 0.0491<br>VM C57Bl6/J/mdx +anti-IGF2R Low 4w: *= 0.0403<br>VM C57Bl6/J/mdx +anti-IGF2R Low 9w: *= 0.0394<br>VM C57Bl6/J/mdx untreated *= 0.0469<br>VM mdx untreated/mdx +anti-IGF2R Low 9w: *= 0.0108<br>VM mdx +anti-IGF2R High 9w/ mdx +anti-IGF2R Low 9w: **= 0.0010<br>VM mdx +anti-IGF2R High 9w/ mdx +anti-IGF2R Low 4w: *= 0.022<br><br>N=10	Two-way ANOVA
6	A	CD20/vinculin<br>TA mdx + anti-IGF2R Low 4w/C57BL6/J: ***= 0.0002<br>TA mdx + anti-IGF2R High 4w/C57BL6/J: **= 0.0093<br>TA mdx + anti-IGF2R Low 9w/C57BL6/J **= 0.0021<br>TA mdx untreated/C57BL6/J **= 0.0011<br>TA mdx untreated/ mdx + anti-IGF2R High 9w ***= 0.0010<br>TA mdx untreated/ mdx + anti-IGF2R Low 9w *=0.027<br>pSer+pThr/CD20<br>TA mdx + anti-IGF2R Low 4w/C57BL6/J: ****<0.0001<br>TA mdx + anti-IGF2R High 4w/C57BL6/J: ****<0.0001<br>TA mdx + anti-IGF2R Low 9w/C57BL6/J *= 0.0465<br>TA mdx + anti-IGF2R High 9w/C57BL6/J: **= 0.0054<br>TA mdx untreated/C57BL6/J **= 0.0018<br><br>CD20/vinculin<br>VM mdx + anti-IGF2R Low 4w/C57BL6/J: **=0.0039<br>VM mdx + anti-IGF2R High 4w/C57BL6/J: ****<0.0001<br>VM mdx + anti-IGF2R Low 9w/C57BL6/J ****<0.0001<br>VM mdx + anti-IGF2R High 9w/C57BL6/J: ***= 0.0008<br>VM mdx untreated/C57BL6/J ****<0.0001<br>pSer+pThr/CD20<br>VM mdx + anti-IGF2R Low 4w/C57BL6/J: ****<0.0001<br>VM mdx + anti-IGF2R High 4w/C57BL6/J: ****<0.0001<br>VM mdx + anti-IGF2R Low 9w/C57BL6/J ***=0.0002<br>VM mdx + anti-IGF2R High 9w/C57BL6/J: ****<0.0001	One-way ANOVA

		VM C57Bl6/J/mdx untreated: ****<0.0001<br><br>N=10	
6	B	pIGF1R/IGF1R<br>TA mdx untreated/C57BL6/J: ****<0.0001<br>TA C57Bl6/J/mdx +anti-IGF2R High 4w: ****<0.0001<br>TA C57Bl6/J/mdx +anti-IGF2R High 9w: ****<0.0001<br>TA C57Bl6/J/mdx +anti-IGF2R Low 9w: ****<0.0001<br><br>VM mdx untreated/C57BL6/J: ****<0.0001<br>VM C57Bl6/J/mdx +anti-IGF2R High 4w: ****<0.0001<br>VM C57Bl6/J/mdx +anti-IGF2R High 9w: ****<0.0001<br>VM C57Bl6/J/mdx +anti-IGF2R Low 4w: ****<0.0001<br>VM C57Bl6/J/mdx +anti-IGF2R Low 9w: ****<0.0001<br><br>N=10	One-way ANOVA
7	C	% CD146/image<br>TA mdx + anti-IGF2R Low 4w/mdx untreated: ****<0.0001<br>TA mdx + anti-IGF2R High 4w/mdx untreated: ****<0.0001<br>TA mdx + anti-IGF2R High 9w/mdx untreated: ****<0.0001<br><br>% NG2/image<br>TA mdx + anti-IGF2R Low 4w/mdx untreated: ****<0.0001<br>TA mdx + anti-IGF2R High 4w/mdx untreated: ***= 0.00025<br>TA mdx + anti-IGF2R High 9w/mdx untreated: ****<0.0001<br><br>N=10	One-way ANOVA
7	D	CD146/ β-actin<br>TA mdx + anti-IGF2R High 4w/mdx untreated: ****<0.0001<br>TA mdx + anti-IGF2R High 9w/mdx untreated: **= 0.0053<br>TA mdx + anti-IGF2R Low 9w/mdx untreated: ***= 0.00069<br>NG2/ β-actin<br>TA mdx + anti-IGF2R High 4w/mdx untreated: *= 0.0415<br>TA mdx + anti-IGF2R High 9w/mdx untreated: *= 0.0236<br>TA mdx + anti-IGF2R Low 9w/mdx untreated: ***= 0.00065<br><br>CD146/ β-actin<br>VM mdx +anti-IGF2R High 4w/untreated mdx: ****<0.0001<br>VM mdx +anti-IGF2R High 9w/untreated mdx: ****<0.0001<br>VM mdx +anti-IGF2R Low 4w/untreated mdx: ***= 0.00079<br>VM mdx +anti-IGF2R Low 9w/untreated mdx: ****<0.0001<br>NG2/ β-actin<br>VM mdx +anti-IGF2R High 4w/untreated mdx: ****<0.0001<br>VM mdx +anti-IGF2R High 9w/untreated mdx: ****<0.0001<br>VM mdx +anti-IGF2R Low 4w/untreated mdx: ***= 0.00055<br>VM mdx +anti-IGF2R Low 9w/untreated mdx: ****<0.0001<br><br>N=10	One-way ANOVA
EV1	A	CD20/ β-actin<br>Untreated/ShCD20: ****<0.0001<br>OE CD20/Untreated: ****<0.0001<br>OE CD20/ShCD20: ****<0.0001<br><br>N=5	One-way ANOVA
EV2	B	p-CamKII/CamKII<br>ShCD20 + anti-IGF2R C2C12 2 days DM/untreated C2C12 2 days DM: ****<0.0001<br>ShCD20 + anti-IGF2R C2C12 2 days DM/untreated C2C12 2 days DM: ***=0.0002<br>P.M. anti-IGF2R C2C12/untreated C2C12: ***= 0.00016<br>anti-IGF2R C2C12 2 days DM/ C2C12 2 days DM: ****<0.0001	Two-way ANOVA

		anti-IGF2R C2C12 4 days DM/ C2C12 4 days DM: ****<0.0001<br>anti-IGF2R C2C12 6 days DM/ C2C12 6 days DM: ****<0.0001<br>P.M. anti-IGF2R C2C12 /untreated Sh-CD20 C2C12: ***= 0.0004<br>anti-IGF2R C2C12 2 days DM/ Sh-CD20 C2C12 2 days DM: ****<0.0001<br>anti-IGF2R C2C12 4 days DM/ Sh-CD20 C2C12 4 days DM: ****<0.0001<br>anti-IGF2R C2C12 6 days DM/ Sh-CD20 C2C12 6 days DM: ****<0.0001	
EV2	C	CalcA/vinculin<br>ShCD20 + anti-IGF2R C2C12 2 days DM/untreated C2C12 2 days DM: **= 0.0034<br>ShCD20 + anti-IGF2R C2C12 4 days DM/untreated C2C12 4 days DM: ****<0.0001<br>ShCD20 + anti-IGF2R C2C12 6 days DM/untreated C2C12 6 days DM: **= 0.0058<br>anti-IGF2R C2C12 4 days DM/ C2C12 4 days DM: ****<0.0001<br>anti-IGF2R C2C12 6 days DM/ C2C12 6 days DM: *= 0.0102<br>anti-IGF2R C2C12 4 days DM/ Sh-CD20 C2C12 4 days DM: ****<0.0001<br>anti-IGF2R C2C12 6 days DM/ Sh-CD20 C2C12 6 days DM: *= 0.0299	Two-way ANOVA
EV2	E	Cyt/Nuc<br>Untreated/S.D. + Ca^{2+} 5mM: **= 0.0072<br>Untreated/S.D. + anti-IGF2R + Ca^{2+} 5mM: ****<0.0001<br>S.D. + Ca^{2+} 5mM/S.D. + anti-IGF2R + Ca^{2+} 5mM: **= 0.0090<br><br>N=3	One-way ANOVA
EV2	F	Myod/vinculin<br>S.D. + anti-IGF2R + Ca^{2+} 5mM/S.D. + Ca^{2+} 5mM: ***= 0.00044<br>S.D. + anti-IGF2R + Ca^{2+} 5mM/anti-IGF2R + Ca^{2+} 5mM: ***= 0.00092<br>S.D. + anti-IGF2R + Ca^{2+} 5mM/mdx untreated: ***= 0.0006<br>S.D. + Ca^{2+} 5mM/anti-IGF2R + Ca^{2+} 5mM: **= 0.0079<br><br>N=3	One-way ANOVA
EV3	B	Fluo4+ (%)<br>C2C12 myoblasts + anti-IGF2R/C2C12: *= 0.0433<br><br>Mean Fluo<br>C2C12 myoblasts + anti-IGF2R/C2C12: *= 0.035	One-way ANOVA
	E	Fluo4+ (%)<br>C2C12 myoblasts-ShCD20/C2C12: *= 0.0317<br>C2C12 myoblasts-ShCD20 + anti-IGF2R/C2C12: **= 0.0087	One-way ANOVA
	G	C2C12 anti-IGF2R/C2C12: *= 0.0410	One-way ANOVA
	L	ORAI-1<br>Untreated/S.D. + Ca^{2+} 5mM: **= 0.0057<br>Untreated/S.D. + anti-IGF2R + Ca^{2+} 5mM: ***= 0.00063<br>S.D. + Ca^{2+} 5mM/S.D. + anti-IGF2R + Ca^{2+} 5mM: **= 0.0074	One-way ANOVA
EV4	B	Ratio pSer-pThr/CD20<br>S.D. C2C12 myoblasts/untreated C2C12 myoblasts: ****<0.0001<br>S.D. C2C12 myoblasts/S.D. C2C12 ShC20 myoblasts: ****<0.0001<br>S.D. + anti-IGF2R + C2C12 myoblasts/untreated C2C12 myoblasts: ****<0.0001<br>S.D. + anti-IGF2R + C2C12 myoblasts/S.D. + anti-IGF2R + C2C12 ShC20 myoblasts: ****<0.0001<br>C2C12 ShCD20 myoblasts/S.D. + anti-IGF2R + C2C12 ShC20 myoblasts: ****<0.0001<br>C2C12 ShCD20 myoblasts/untreated C2C12 myoblasts: ***= 0.000687<br>Untreated C2C12 myoblasts/C2C12 myoblasts+anti-IGF2R: *= 0.0323	One-way ANOVA
