# Supplementary material for: Blockade of IGF2R improves muscle regeneration and ameliorates Duchenne muscular dystrophy
Source: EMBO Mol Med. 2019 Dec 2;12(1):e11019. doi: 10.15252/emmm.201911019 (PMC6949491; doi:10.15252/emmm.201911019)
Supplement: Supplementary file 2 — Expanded View Figures PDF [file EMMM-12-e11019-s002.pdf]

Expanded View Figures

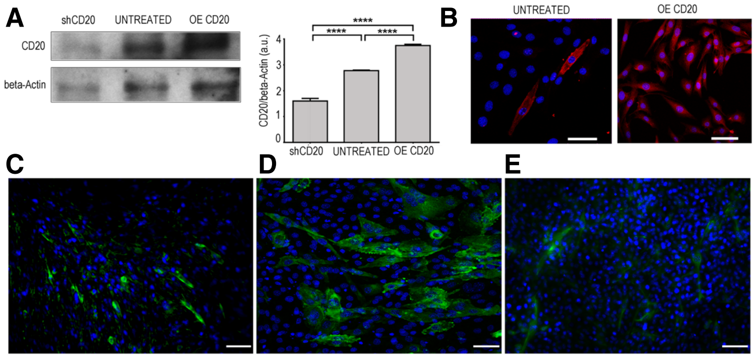

Figure EV1. The effect of CD20 modulation in C2C12 and 3T3 myoblasts.

A Knockdown (shCD20) and over-expression (OE) of CD20 was tested by WB analysis. Densitometry analysis of data expressed as the CD20/β-actin ratio in arbitrary units. One-way ANOVA. ****$P < 0.0001$ ($n = 5$ independent experiments). All values are expressed as the mean ± SEM.

B Immunofluorescence showing CD20 expression in untreated and CD20 over-expressing C2C12 myoblasts. Scale bars = 75 μm.

C–E 3T3 mouse fibroblasts were labelled for anti-IGF1R tyrosine phosphorylation (green) under basal conditions (C), when co-stimulated with IGF2 and anti-IGF2R (D) and in the presence of IGF2 (E). Scale bars = 75 μm.

Source data are available online for this figure.

© 2019 The Authors

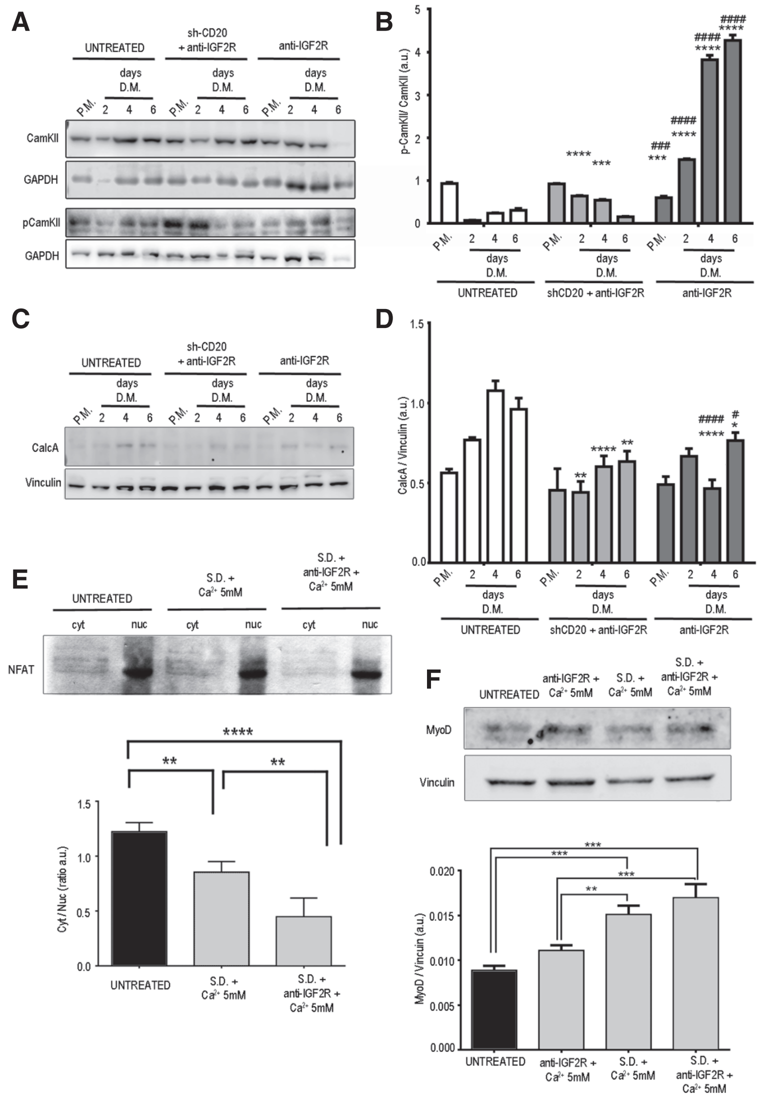

Figure EV2. Calcineurin signalling and NFAT activation in myoblasts treated with anti-IGF2R.

A Representative WB analysis of CAMKII, pCAMKII and GAPDH levels in total protein lysates of untreated and anti-IGF2R-treated C2C12 cells and ShCD20 C2C12 myoblasts grown in proliferation medium (PM) after 2, 4 and 6 days of myogenic differentiation (DM).

B Densitometry analysis of data shown in panel (A) expressed as the ratio of pCAMKII/CAMKII in arbitrary units. Two-way ANOVA test. ***$P < 0.001$; ****$P < 0.0001$ for the comparison with untreated cells at the corresponding time point. $^{\#\#\#}P < 0.001$; $^{\#\#\#\#}P < 0.0001$ for the comparison with C2C12 shCD20 cells at the corresponding time point. Each experiment was performed in triplicate wells. All values are expressed as the mean ± SEM.

C Representative WB analysis of CalcA and vinculin levels in total protein lysates of untreated and anti-IGF2R-treated C2C12 cells and ShCD20 C2C12 myoblasts grown in proliferation medium (PM) and collected after 2, 4 and 6 days of DM.

D Densitometry analysis of data shown in panel (C). Data are expressed as the ratio of CalcA/vinculin in arbitrary units. Two-way ANOVA test. *$P < 0.05$; **$P < 0.01$; ****$P < 0.0001$ for the comparison with untreated cells at the corresponding time point. $^{\#}P < 0.05$; $^{\#\#\#\#}P < 0.0001$ for the comparison with C2C12 shCD20 cells at the corresponding time point. Each experiment was performed in triplicate wells. All values are expressed as the mean ± SEM.

E SDS–PAGE gel and immunoblots showing NFAT expression in cytoplasmic and nuclear fractions of untreated and SD+ 5 mM Ca^{2+} and SD+ 5 mM Ca^{2+}+anti-IGF2R treated C2C12 myoblasts. The image reveals that there was a decrease in the size of the band corresponding to pNFAT in the cytoplasmic samples obtained from SD+ 5 mM Ca^{2+} and SD+ 5 mM Ca^{2+} +anti-IGF2R-treated C2C12 myoblasts. The lower panel shows the quantification of the cytoplasmic/nuclear NFAT signal expressed as a ratio in arbitrary units. The decrease observed in these experiments confirmed the increase in the nuclear localization of NFAT caused by its diminished cytoplasmic phosphorylation. The experiment was performed three times. One-way ANOVA test. **$P < 0.01$; ****$P < 0.0001$. All values are expressed as the mean ± SEM.

F Representative immunoblot images indicating MYOD and vinculin expression in the total cell lysates of untreated and SD+ 5 mM Ca^{2+} and SD+ 5 mM Ca^{2+}+anti-IGF2R and 5 mM Ca^{2+} +anti-IGF2R-treated C2C12 myoblasts; results were quantified and are shown as a ratio expressed in arbitrary units in the lower panel. Experiments were repeated three times. One-way ANOVA test. **$P < 0.01$; ***$P < 0.001$; ****$P < 0.0001$. All values are expressed as the mean ± SEM.

Source data are available online for this figure.

 © 2019 The Authors

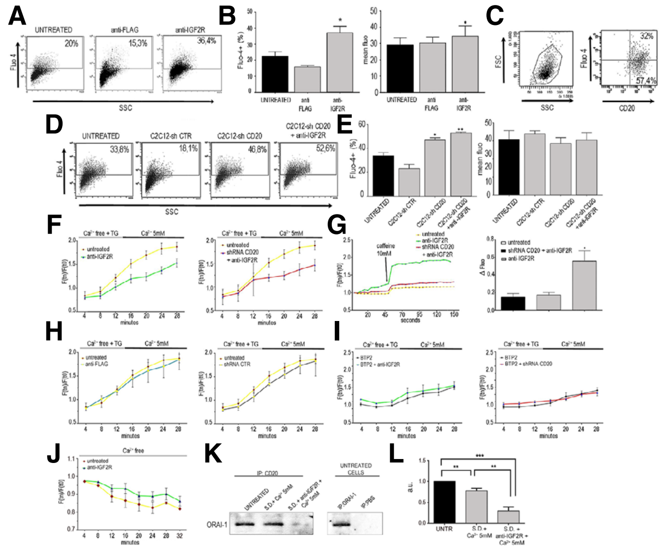

Figure EV3. Intracellular calcium modulation of blockade IGF2R.

A, B Cytofluorimetric analysis of Ca^{2+} and Fluo-4 levels in untreated and treated C2C12 myoblasts with anti-FLAG and anti-IGF2R (A) expressed as the percentage of positive cells and the mean fluorescence (B). One-way ANOVA test. *$P < 0.05$.

C Forward (FSC) and side scatter (SSC) analysis of C2C12 myoblasts stained for Ca^{2+} with Fluo-4 and for anti-CD20.

D, E Fluo-4 and SSC analysis of C2C12-Sh-empty (shCTR) cells, ShCD20-treated C2C12 cells and shCD20 C2C12 myoblasts treated with anti-IGF2R (D). Results are expressed as the percentage of positive cells and the mean fluorescence (E). One-way ANOVA test. *$P < 0.05$; **$P < 0.01$.

F Quantification of Fluo-4 fluorescence in untreated and shCD20-treated C2C12 cells and C2C12 cells treated with anti-IGF2R and anti-FLAG in the presence of free Ca^{2+} and 5 mM Ca^{2+} measured in time-lapse experiments over 28 min. Data are represented as the ratio of fluorescence at Tn vs. T0 over time (min).

G Quantification of Fluo-4 fluorescence in untreated and anti-IGF2R-treated C2C12 cells and C2C12-shCD20 cells stimulated with 10 mM caffeine and subsequently measured in time-lapse experiments over 150 s. Data are represented as the ratio of fluorescence at Tn vs. T0 over time (min). Histograms of data expressed as delta fluorescence before and after caffeine administration. One-way ANOVA test. *$P < 0.05$.

H Quantification of Fluo-4 fluorescence in untreated C2C12 cells, shCD20 C2C12 cells and C2C12 cells treated with anti-FLAG and then measured in time-lapse experiments over 30 min without Ca^{2+} stimulation. Data indicate the ratio of fluorescence-positive cells at Tn vs. T0 over time (min) and are represented as the ratio of fluorescence at Tn vs. T0 over time (min).

I Quantification of Fluo-4 fluorescence in C2C12 + BTP2 cells, C2C12-shCD20 + BTP2 cells and cells treated with anti-IGF2R + BTP2. Results are shown measured in time-lapse experiments performed over 30 min. Data indicate the ratio of fluorescence-positive cells at Tn vs. T0 over time (min) and are represented as the ratio of fluorescence at Tn vs. T0 over time (min).

J Quantification of Fluo-4 in untreated and anti-IGF2R-treated C2C12 cells. Data indicate the ratio of fluorescence-positive cells at Tn vs. T0 over time (min) and are represented as the ratio of fluorescence at Tn vs. T0 over time (min).

K Representative WB of CD20 immunoprecipitation results immunoblotted for ORAI1 in untreated C2C12 cells, store-depleted C2C12 cells and store-depleted + anti-IGF2R-treated C2C12 cells. Representative WB images of ORAI1 immunoprecipitation results immunoblotted for ORAI1 as a positive control and without any specific antibody (PBS) as a negative control.

L Densitometry analysis of the ORAI1 signal expressed in arbitrary units. One-way ANOVA test. **$P < 0.01$; ***$P < 0.001$.

Data information: All experiments were performed in triplicate. All values are expressed as the mean ± SEM.
Source data are available online for this figure.

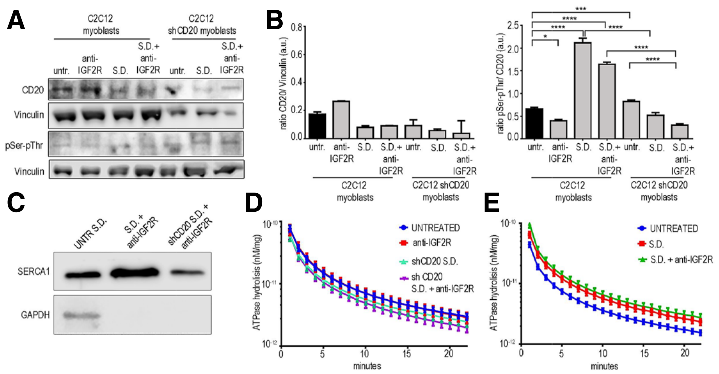

Figure EV4. SERCA modulation of blockade IGF2R.

A Representative WB of CD20 expression and pSer/pThr phosphorylation in C2C12 cells and shCD20-treated C2C12 myoblasts before and after treatment with anti-IGF2R, store depletion (SD) and SD+ anti-IGF2R.

B Densitometric analysis is expressed as the ratio of CD20/vinculin and pSer/pThr/CD20 in arbitrary units. Two-way ANOVA test; $^*P < 0.05$; $^{***}P < 0.001$; $^{****}P < 0.0001$. Each experiment was performed in triplicate wells, and all values are expressed as the mean ± SEM.

C Representative WB images of SERCA1 and GAPDH expression in total lysates of untreated SD C2C12 cells and ER lysates of anti-IGF2R-treated SD C2C12 cells and SD+ shCD20-treated C2C12 myoblasts.

D, E SERCA activity was quantified as ATPase hydrolysis activity vs. min in anti-IGF2R-treated, SD shCD20 C2C12 cells and shCD20 C2C12 + anti-IGF2R cells (D) and in untreated C2C12 cells and SD and SD+ anti-IGF2R-treated C2C12 cells (E). Each experiment was performed in triplicate wells, and all values are expressed as the mean ± SEM.

Source data are available online for this figure.

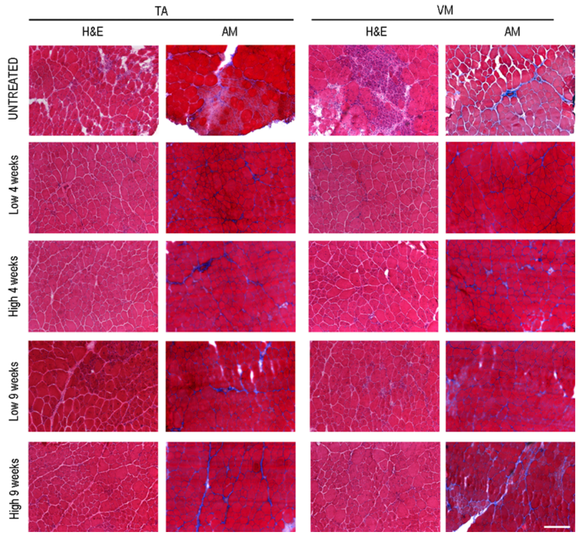

Figure EV5. IGF2R blockade modulates muscular features of *mdx* mice.

Representative haematoxylin and eosin (HE)- and Azan-Mallory (AM)-stained TA and VM tissues of *mdx* mice treated with anti-IGF2R. Scale bar = 200 μm.

 © 2019 The Authors
