# Supplementary figures and images for: Blockade of IGF2R improves muscle regeneration and ameliorates Duchenne muscular dystrophy
Source: EMBO Mol Med. 2019 Dec 2;12(1):e11019. doi: 10.15252/emmm.201911019 (PMC6949491; doi:10.15252/emmm.201911019)

# EV1 A

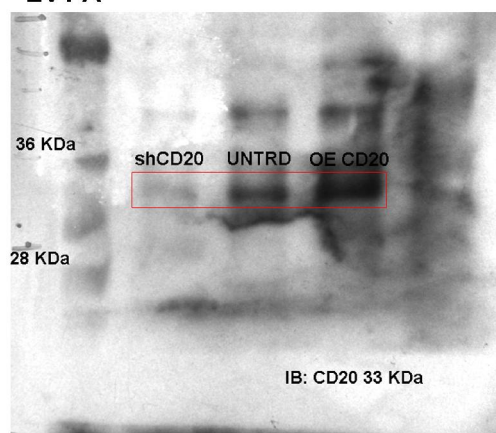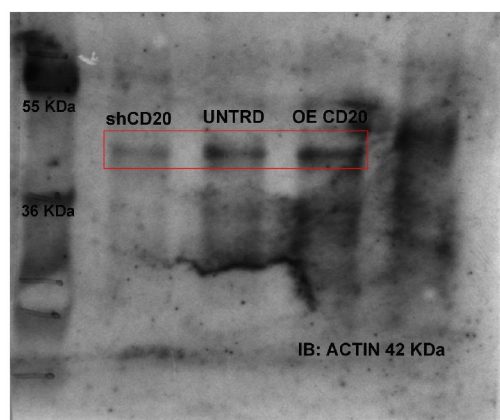

Supplement: Supplementary file 3 — Source Data for Expanded View [file EMMM-12-e11019-s010.zip › EV_source_data/Figure_EV1.pdf]

EV3K

50 KDa

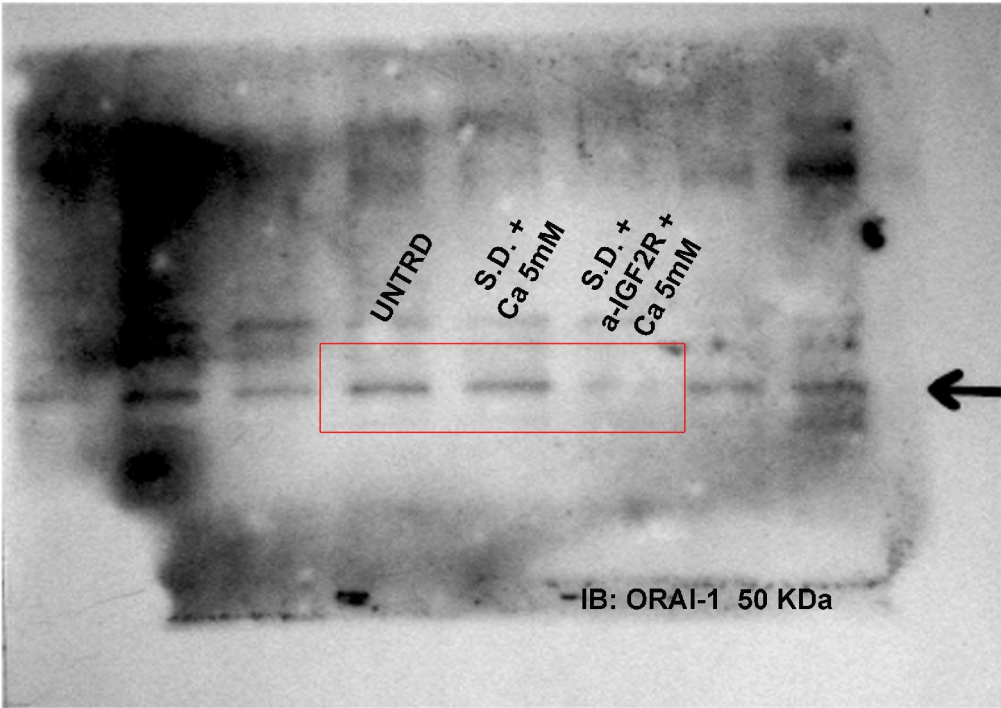

50 KDa

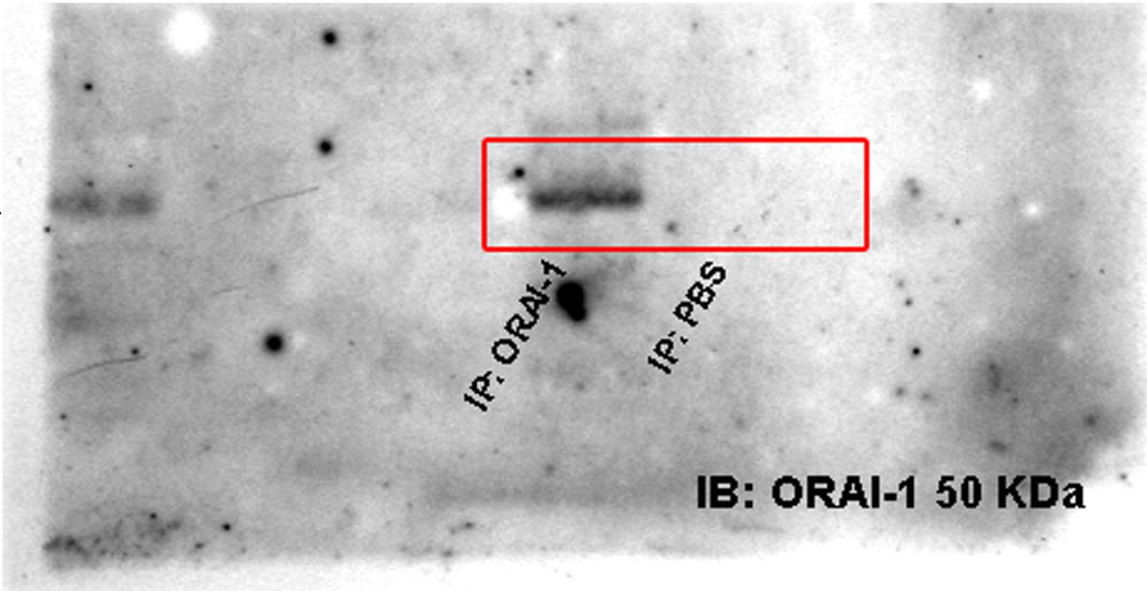

Supplement: Supplementary file 3 — Source Data for Expanded View [file EMMM-12-e11019-s010.zip › EV_source_data/Figure_EV3.pdf]

EV2 A

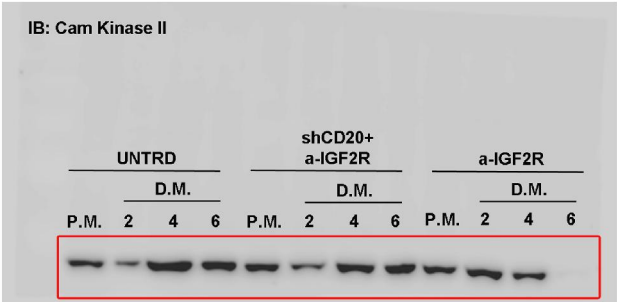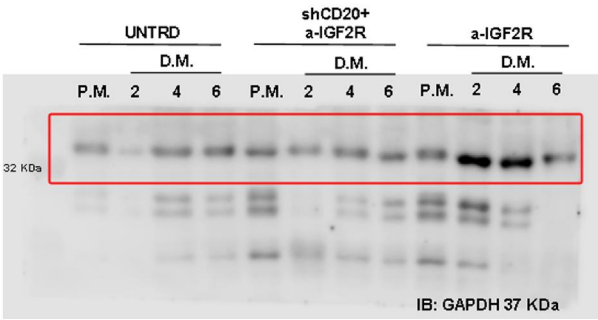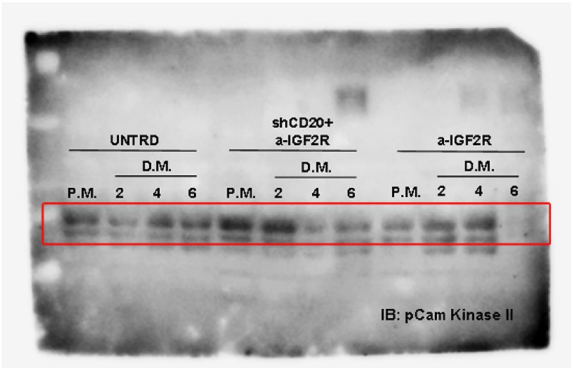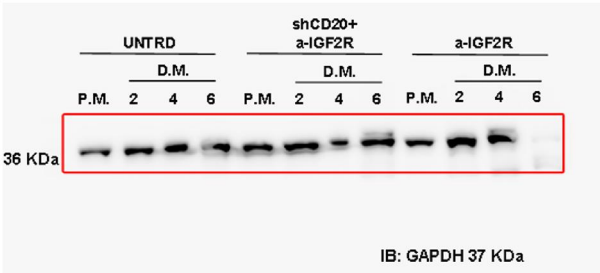

EV2 C

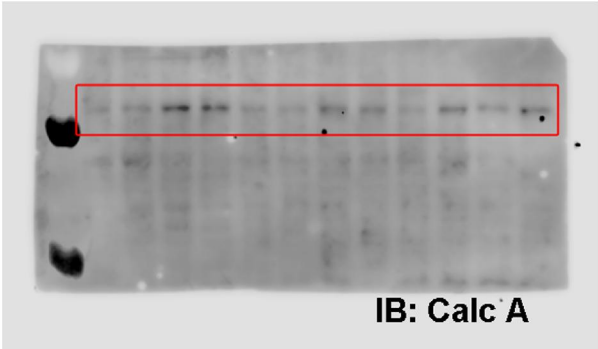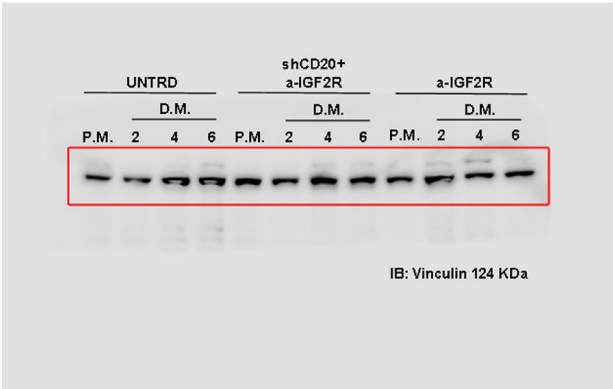

EV2E

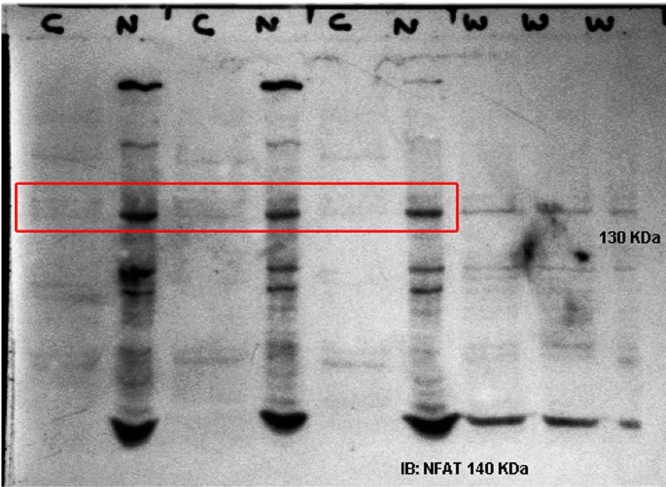

EV2F

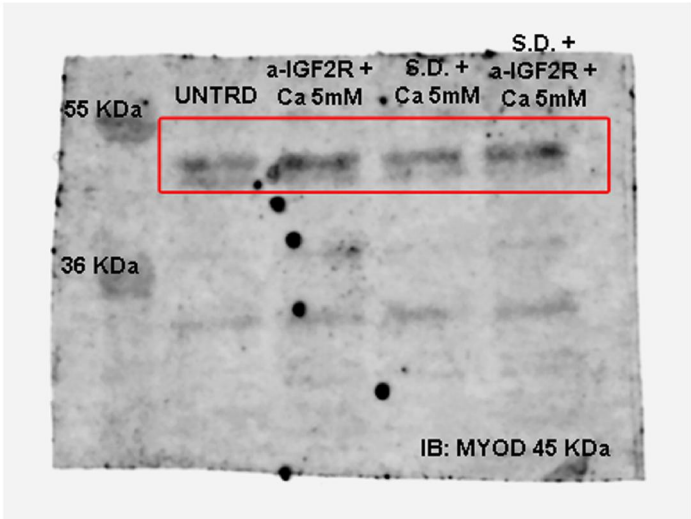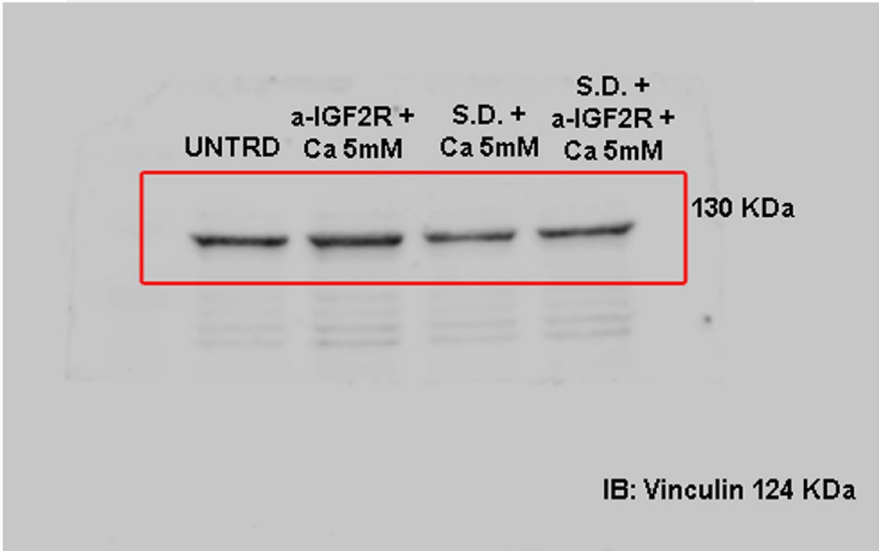

Supplement: Supplementary file 3 — Source Data for Expanded View [file EMMM-12-e11019-s010.zip › EV_source_data/Figure_EV2.pdf]

EV4A

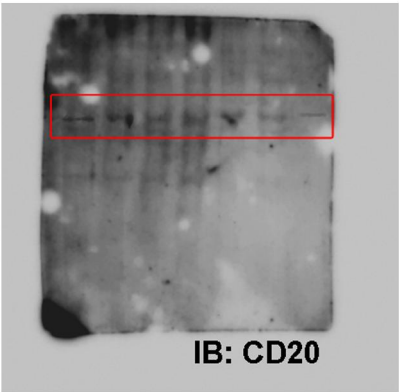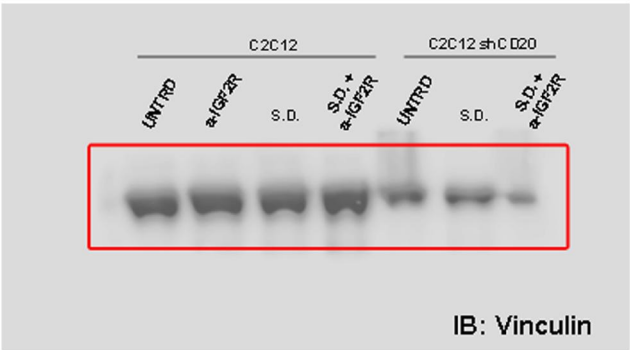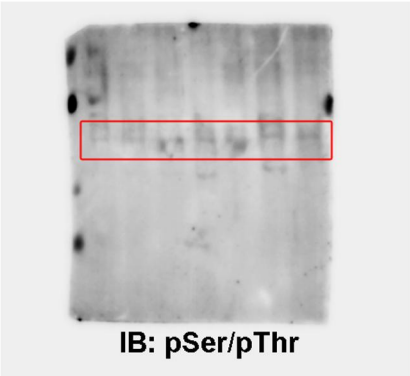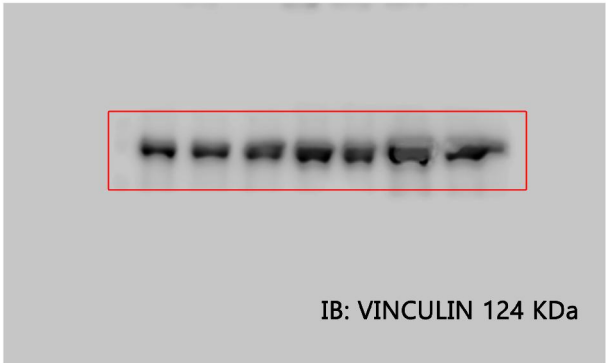

EV4C

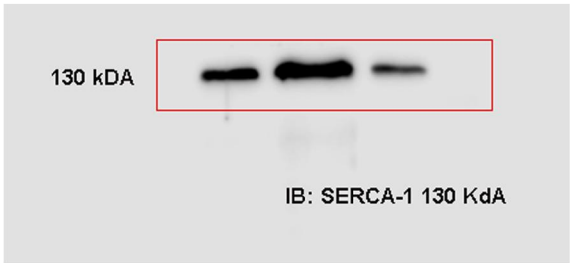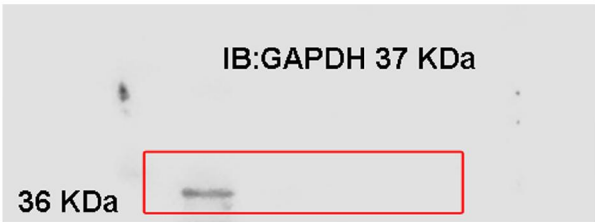

Supplement: Supplementary file 3 — Source Data for Expanded View [file EMMM-12-e11019-s010.zip › EV_source_data/Figure_EV4.pdf]

1A

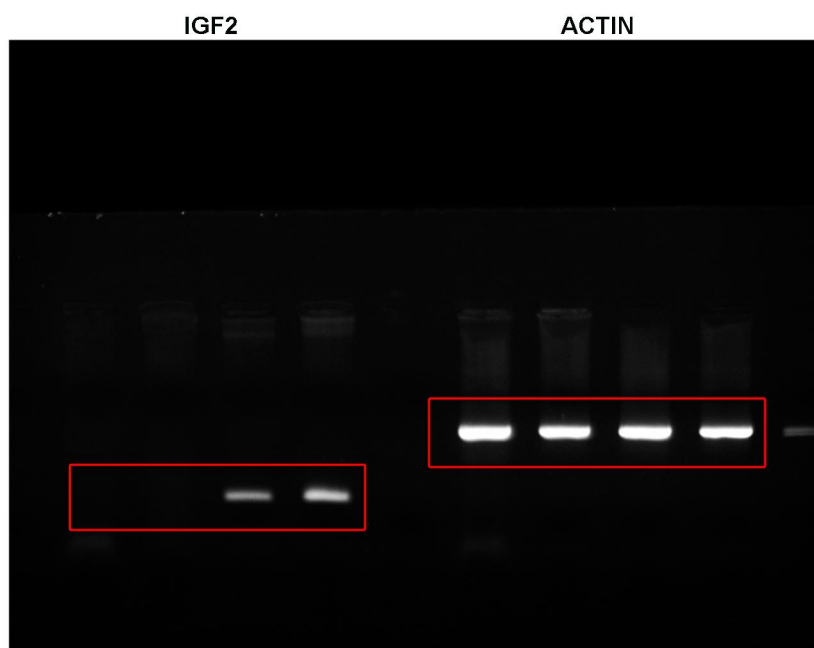

IGF1Rb

IGF1

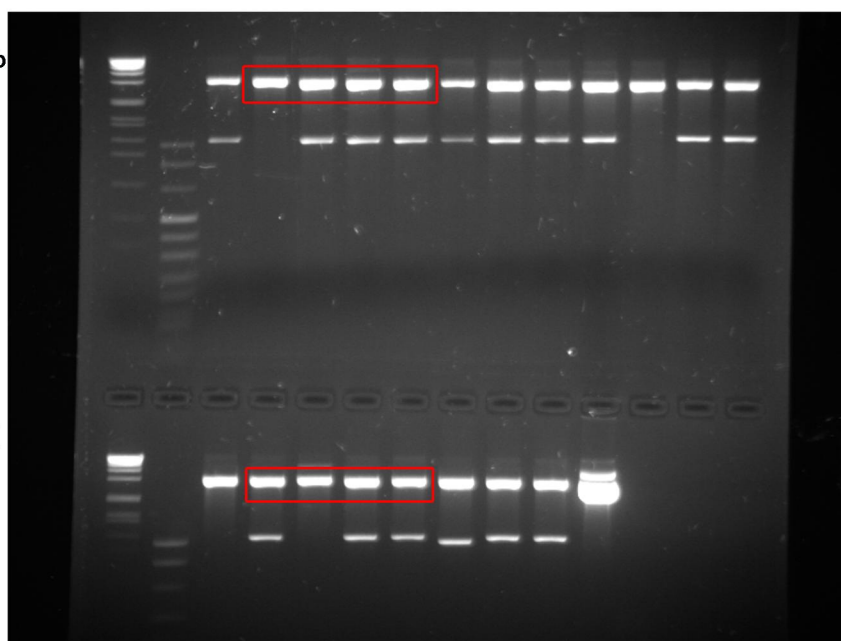

1C

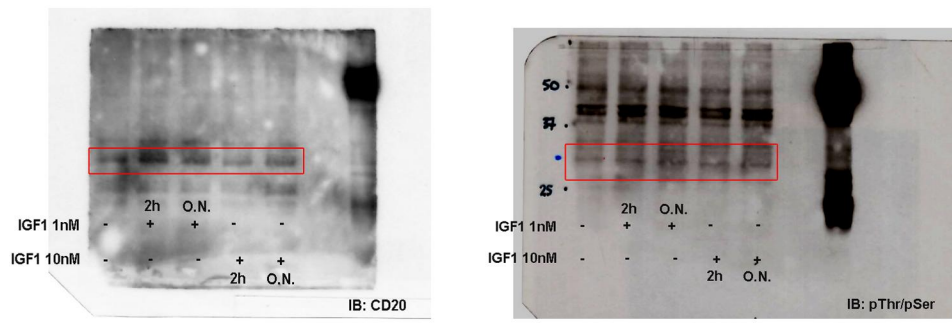

1D

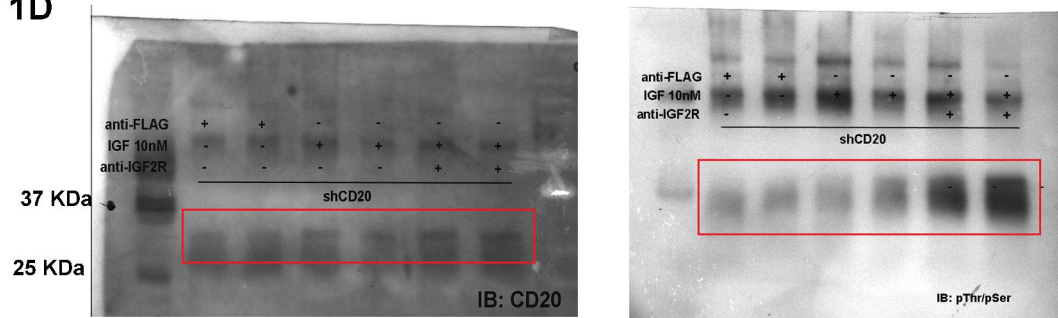

1E

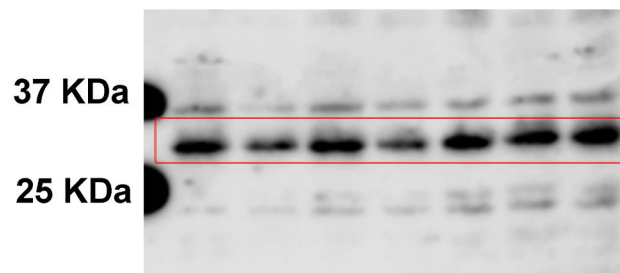

IP CD20: CD20 33 KDa

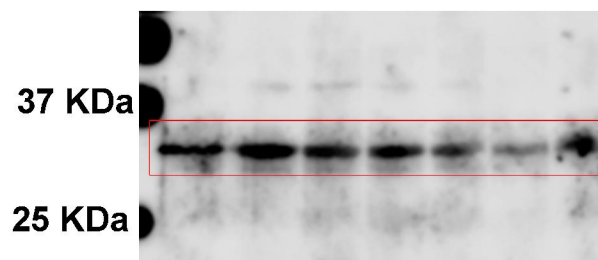

IP CD20: pSer/pThr 33 KDa

1 F\_G

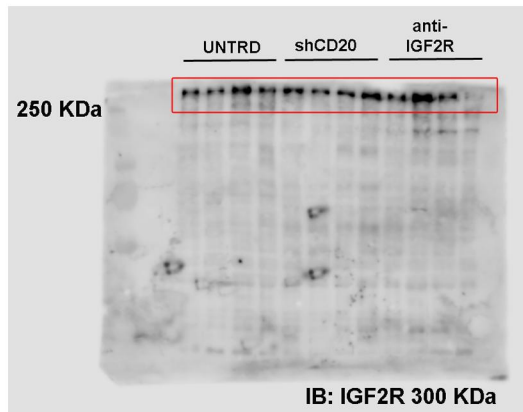

1 F

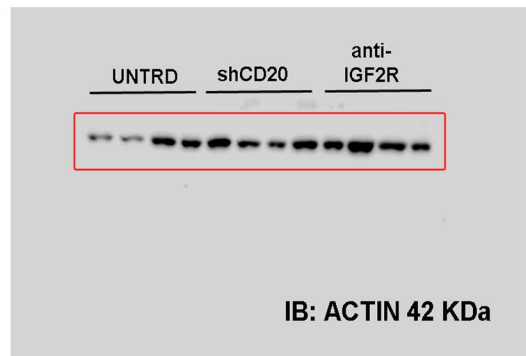

1G

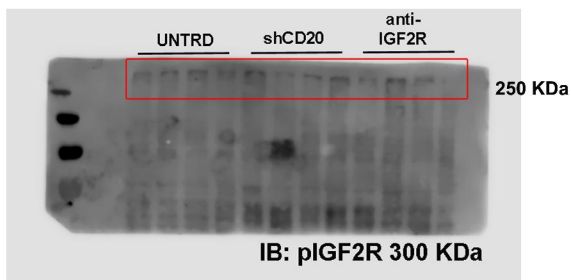

1H

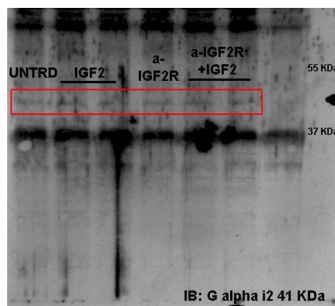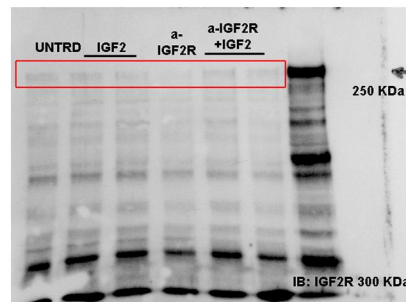

1I

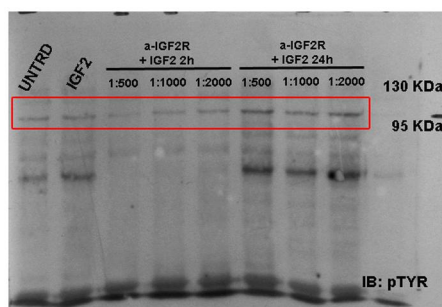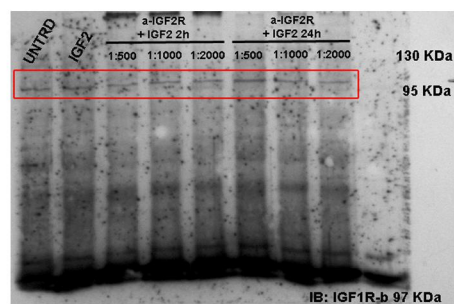

Supplement: Supplementary file 5 — Source Data for Figure 1 [file EMMM-12-e11019-s003.pdf]

2E

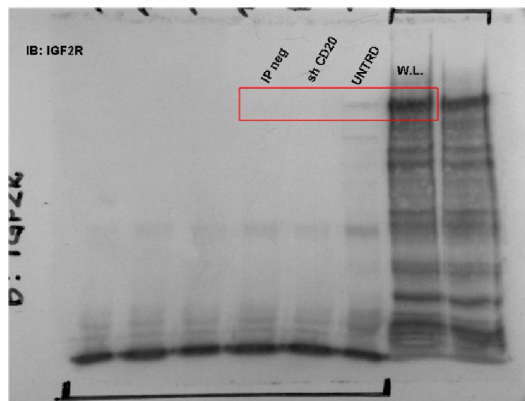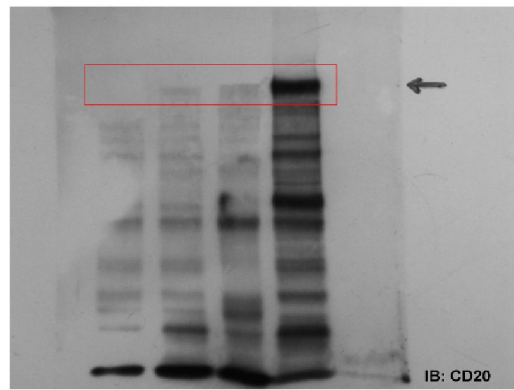

2H

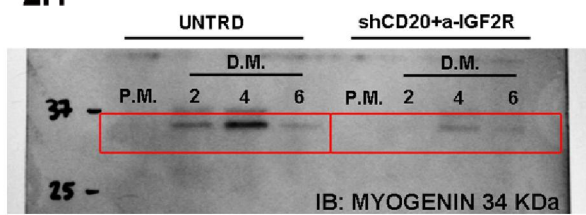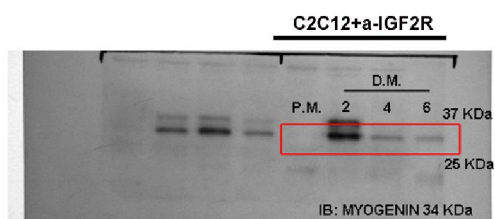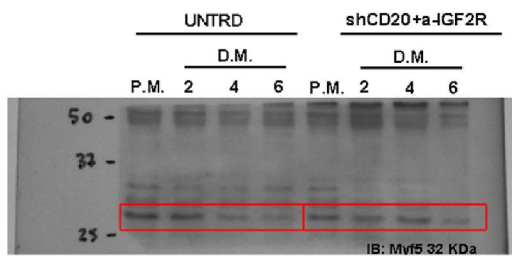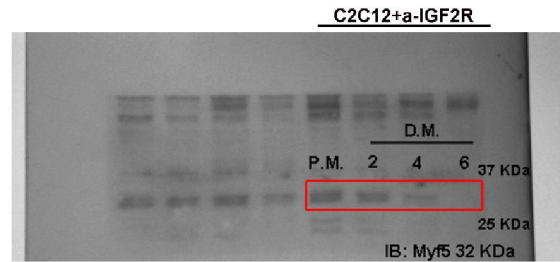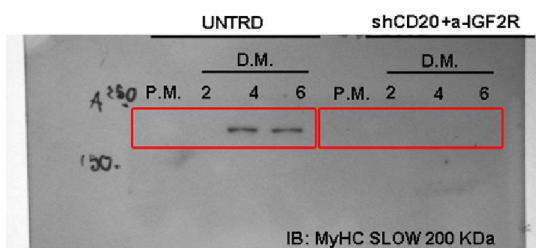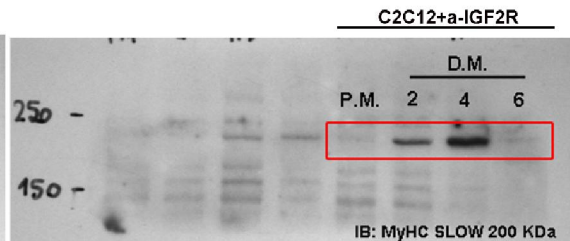

2H

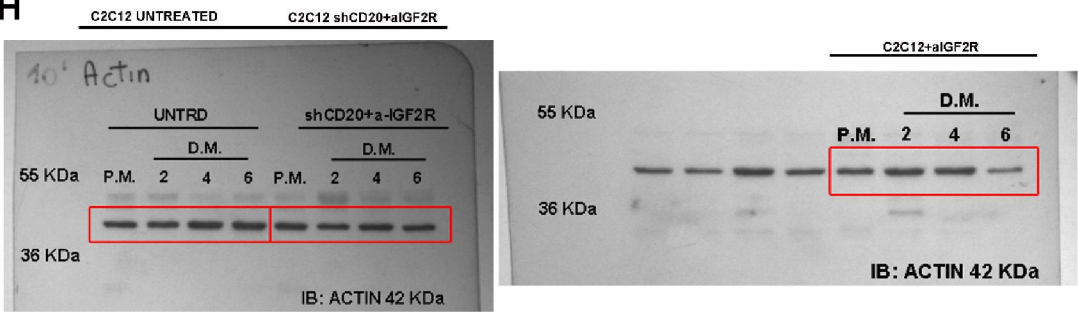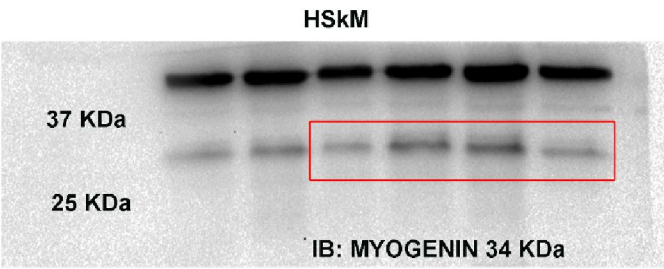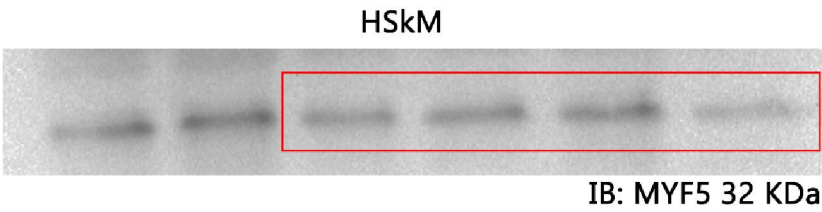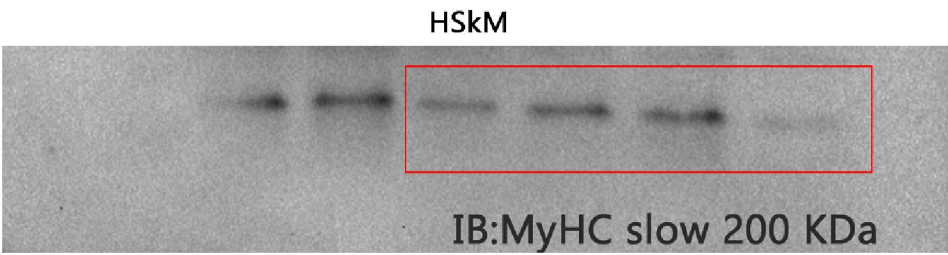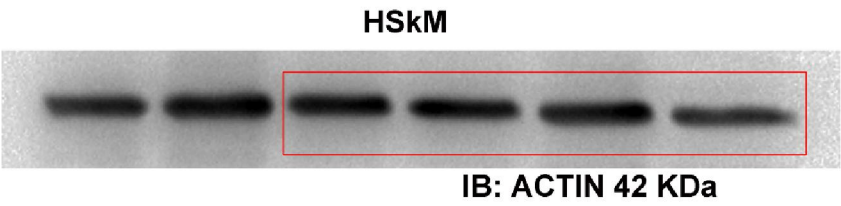

Supplement: Supplementary file 6 — Source Data for Figure 2 [file EMMM-12-e11019-s004.pdf]

3C

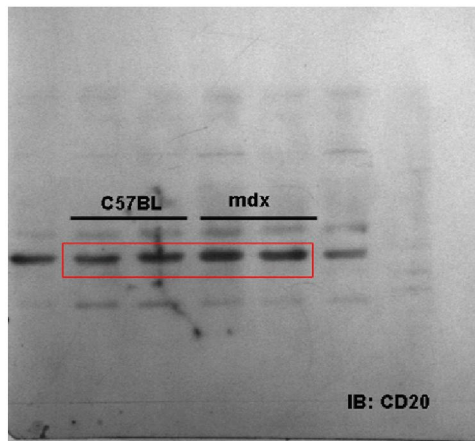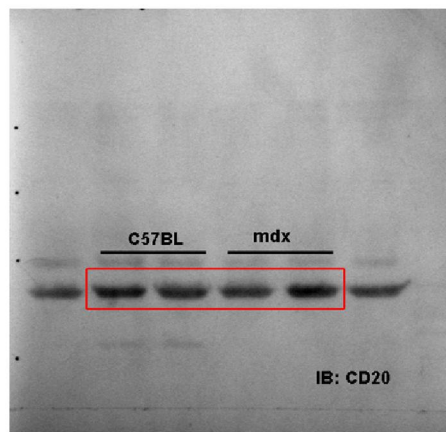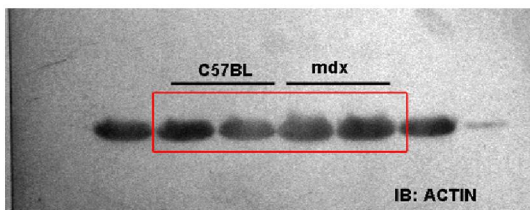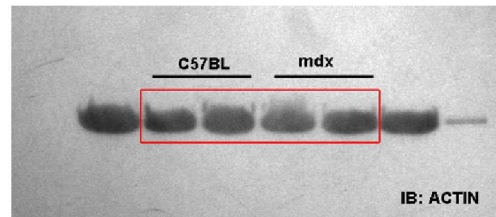

3D

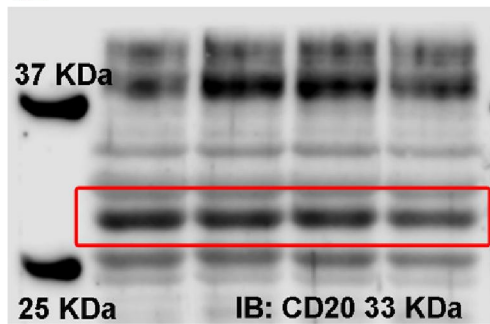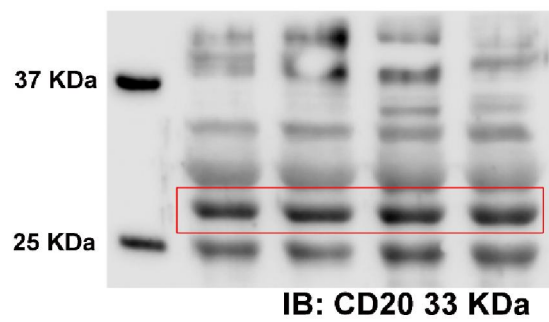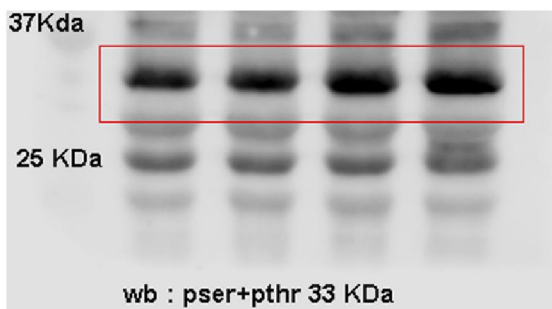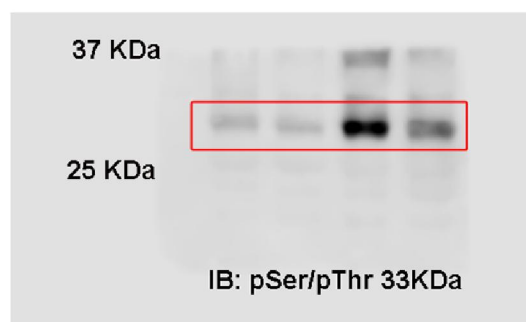

**3D**

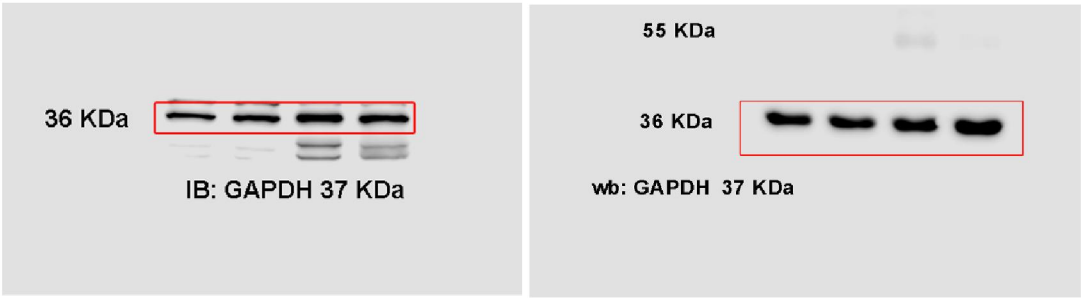

**3E**

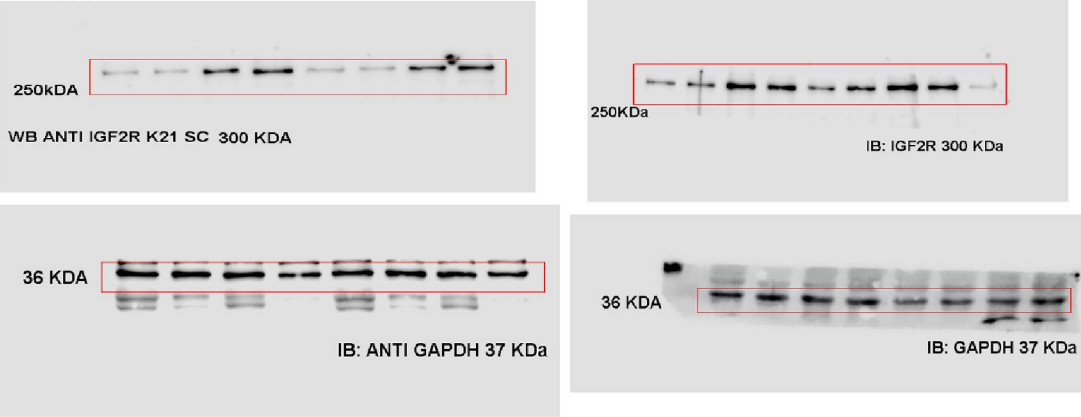

**3F**

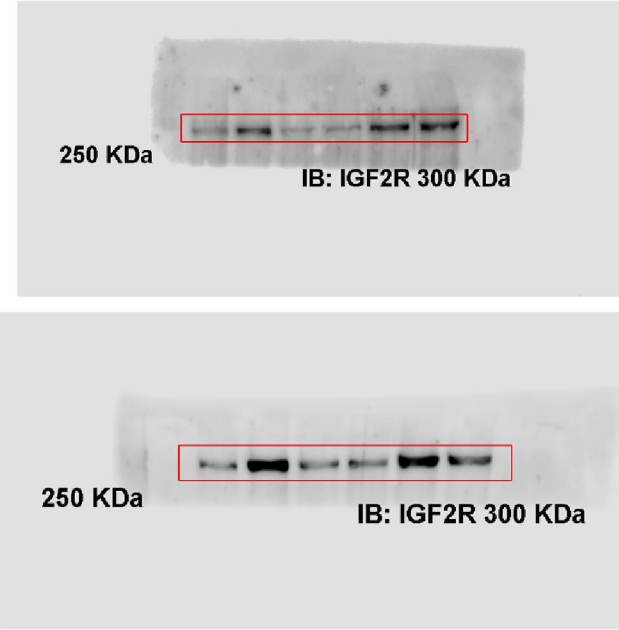

Supplement: Supplementary file 7 — Source Data for Figure 3 [file EMMM-12-e11019-s005.pdf]

**4C**

**Untreated**

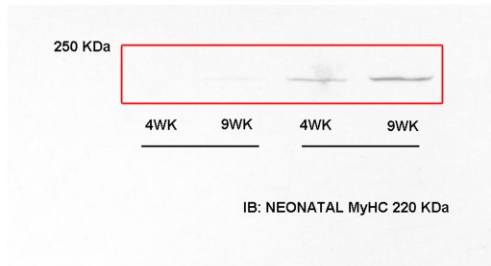

**Untreated**

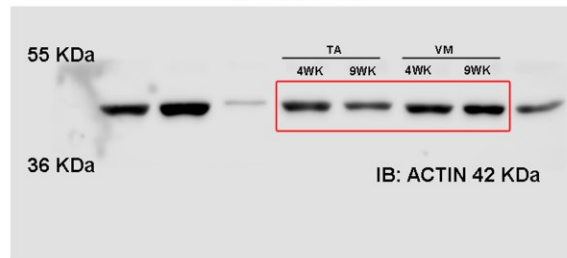

**Low anti-IGF2R**

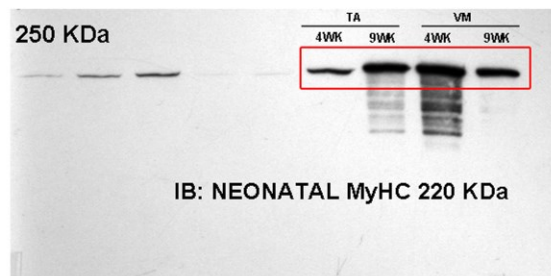

**Low anti-IGF2R**

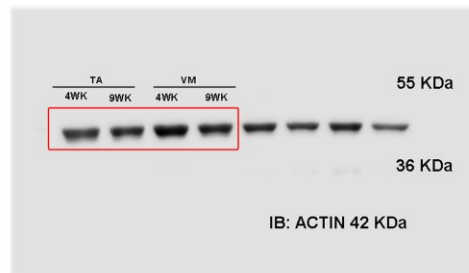

**High anti-IGF2R**

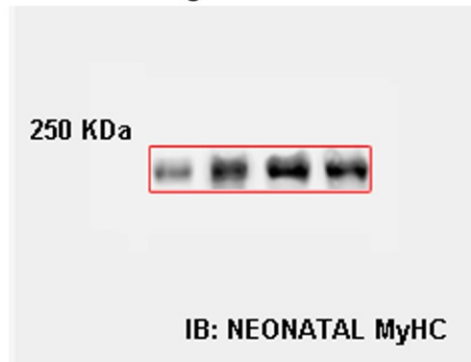

**High anti-IGF2R**

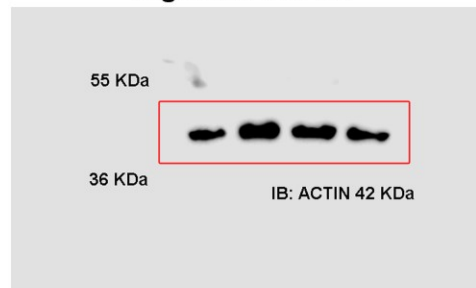

Supplement: Supplementary file 8 — Source Data for Figure 4 [file EMMM-12-e11019-s006.pdf]

5A

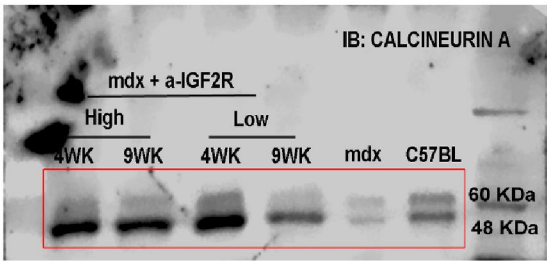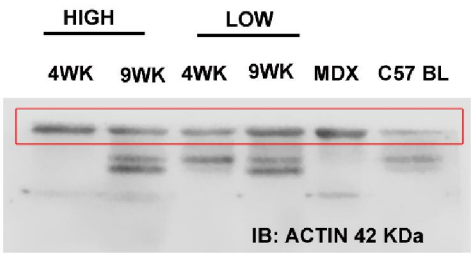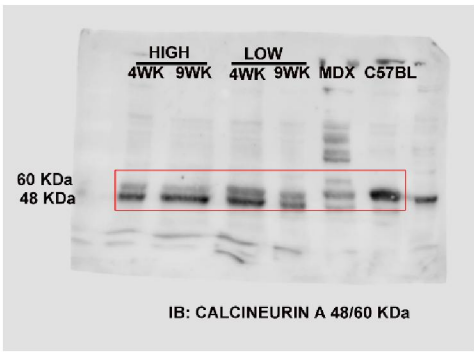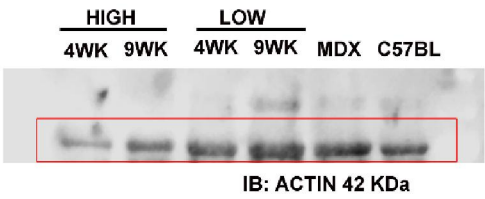

5B

IB: pCam Kinase II 45,70 KDa

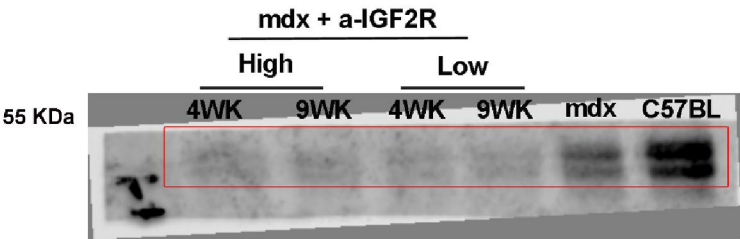

IB: pCam Kinase II 45,70 KDa

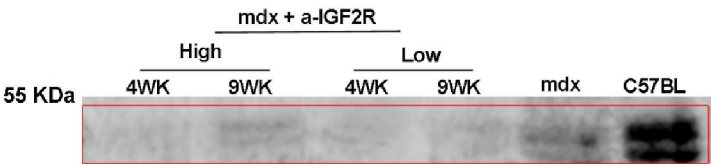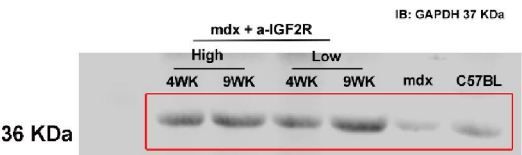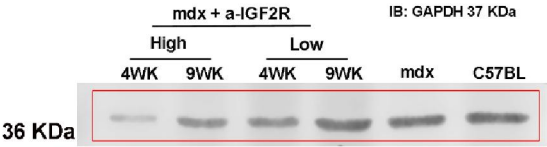

5B

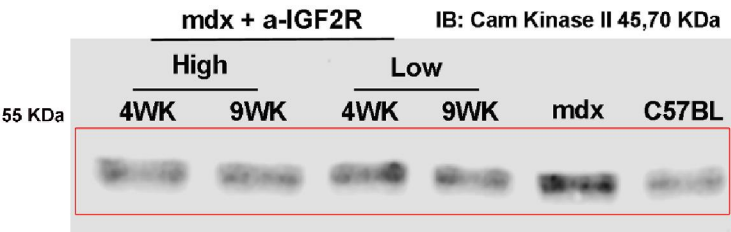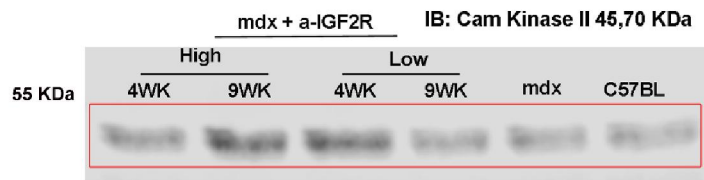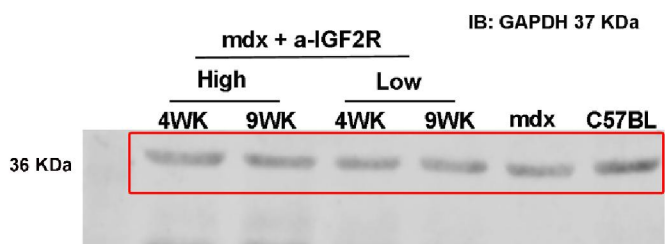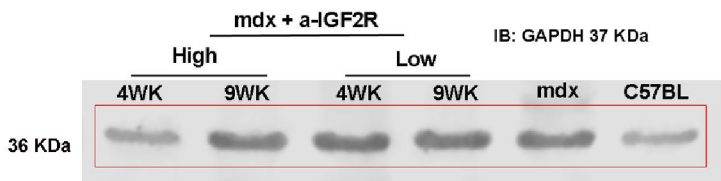

5C

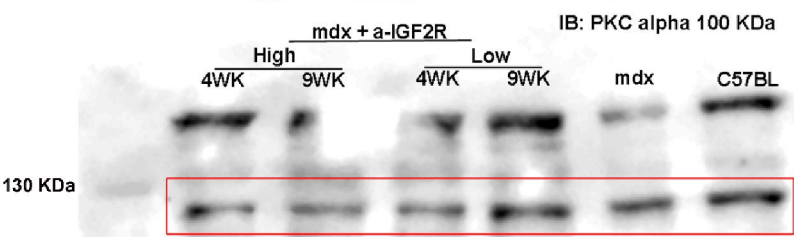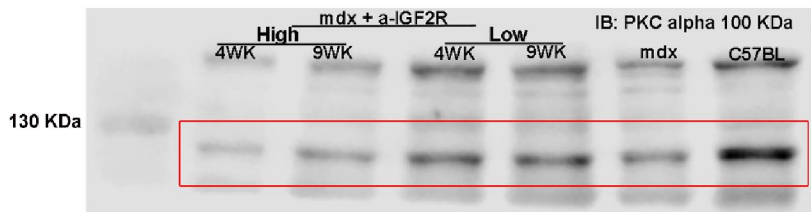

5C

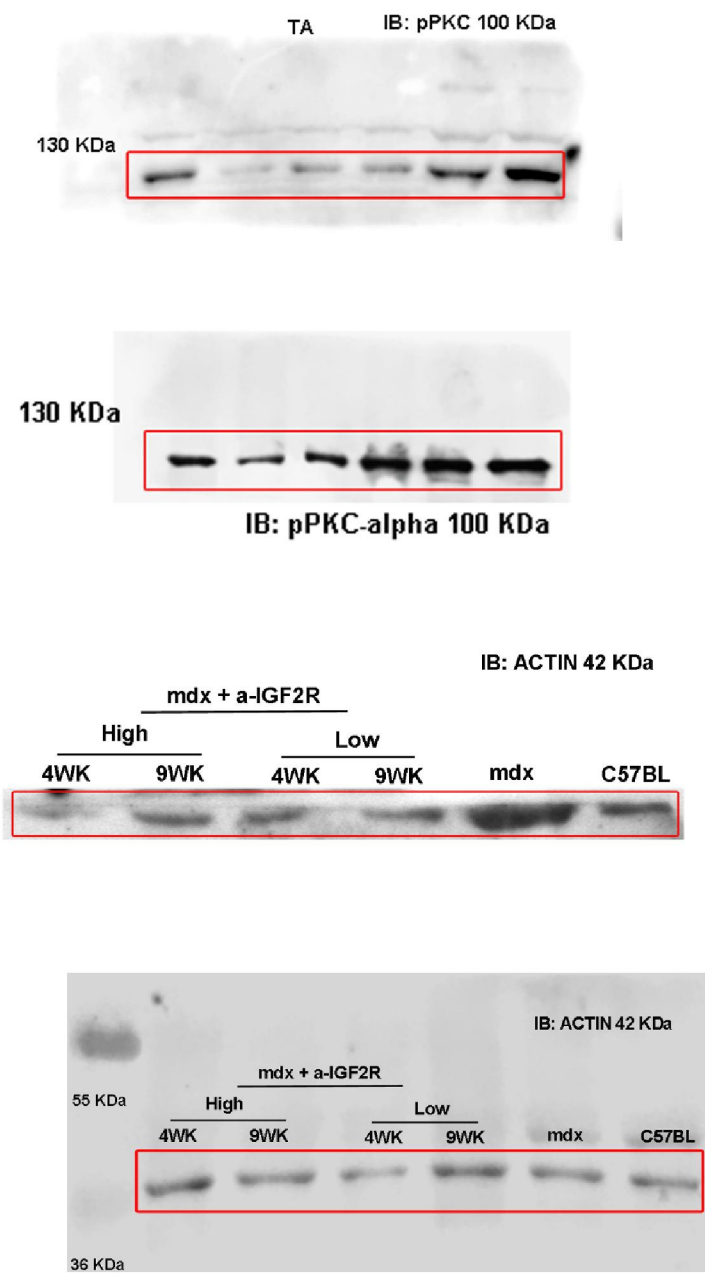

Supplement: Supplementary file 9 — Source Data for Figure 5 [file EMMM-12-e11019-s007.pdf]

**6A**

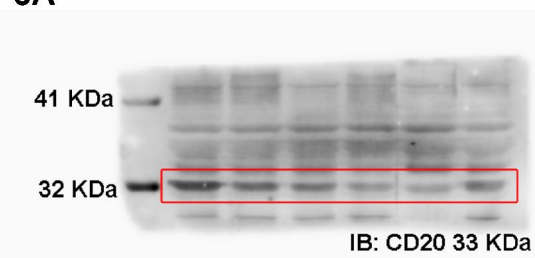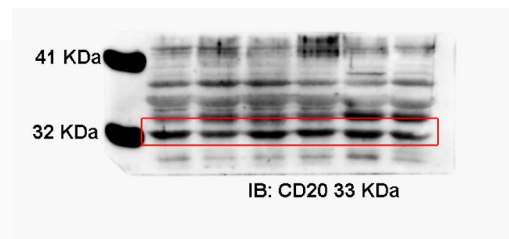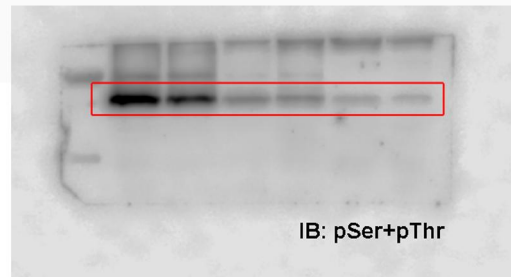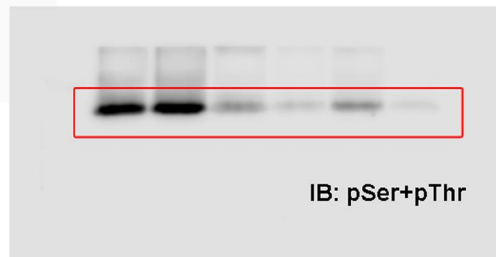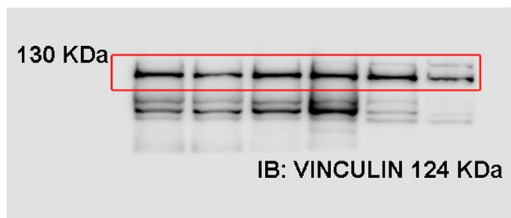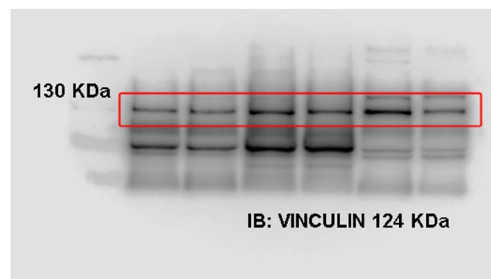

**6B**

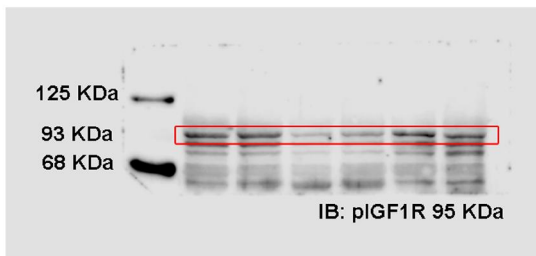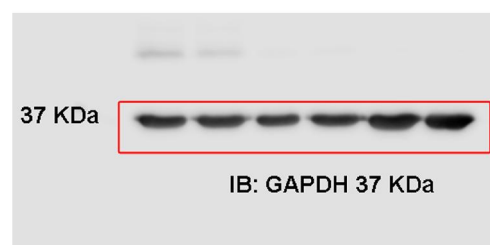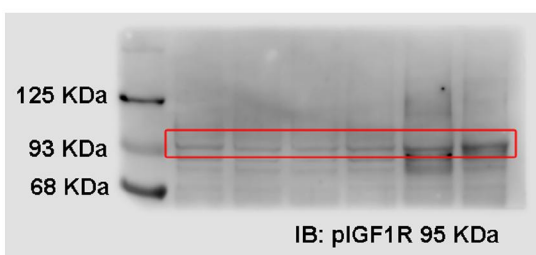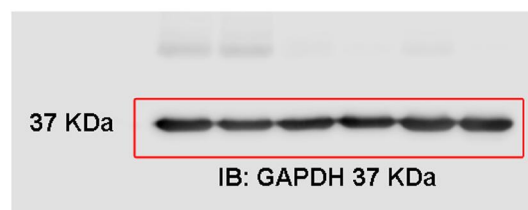

**6 B**

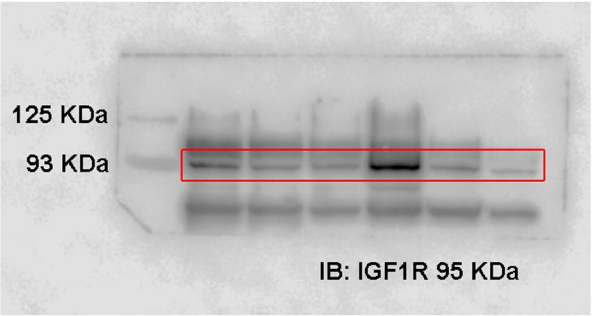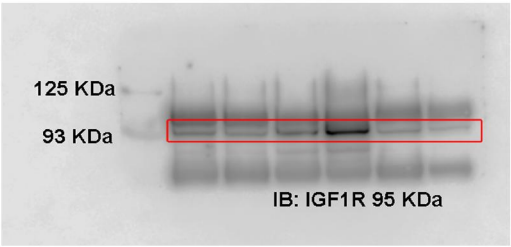

Supplement: Supplementary file 10 — Source Data for Figure 6 [file EMMM-12-e11019-s008.pdf]

7 D

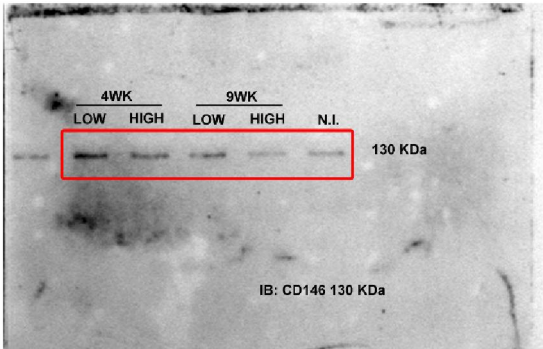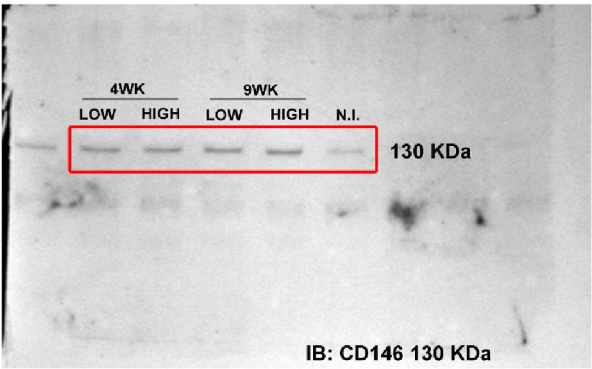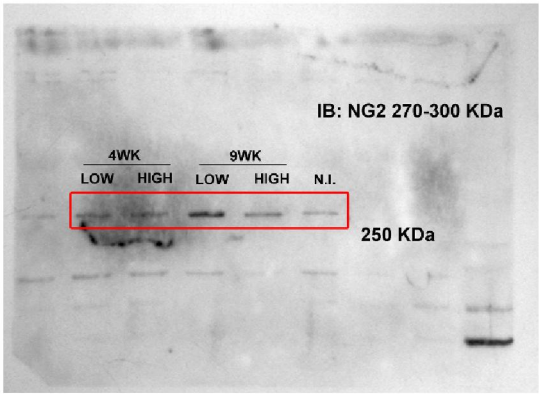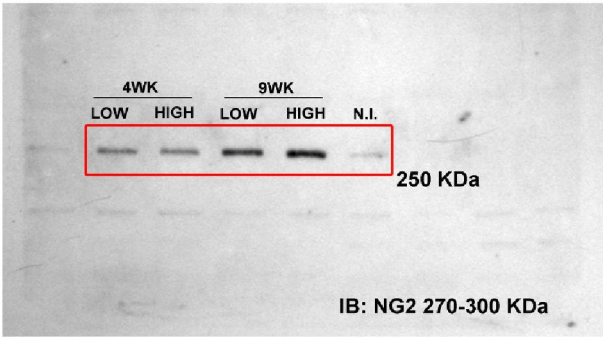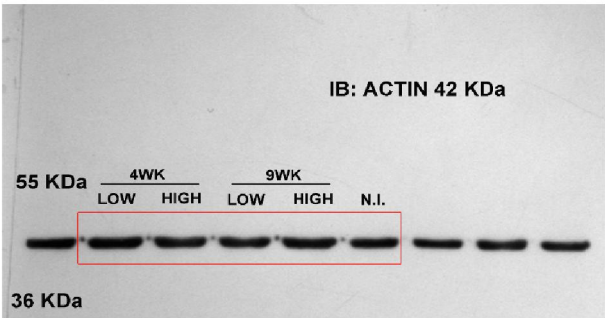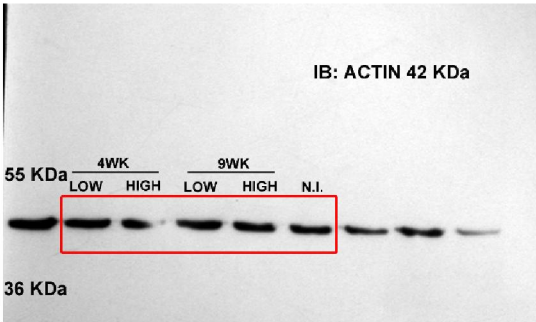

Supplement: Supplementary file 11 — Source Data for Figure 7 [file EMMM-12-e11019-s009.pdf]
